# Educational level, clinical outcomes and quality of care in a Swiss cohort of patients with acute coronary syndromes

**DOI:** 10.1111/eci.70097

**Published:** 2025-07-26

**Authors:** Maëlle Achard, Cédric Follonier, Evelyne Fournier, David Carballo, Mattia Branca, Dik Heg, David Nanchen, Lorenz Räber, Roland Klingenberg, Stephan Windecker, Thomas F. Lüscher, Christian M. Matter, Nicolas Rodondi, François Mach, Baris Gencer

**Affiliations:** ^1^ Cardiology Department Geneva University Hospitals Geneva Switzerland; ^2^ Institute of Global Health University of Geneva Geneva Switzerland; ^3^ Institute of Social and Preventive Medicine, and Clinical Trials Unit, Department of Clinical Research University of Bern Bern Switzerland; ^4^ Department of Ambulatory Care and Community Medicine Lausanne University Lausanne Switzerland; ^5^ Department of Cardiology University Hospital of Bern Bern Switzerland; ^6^ Department of Cardiology University Heart Center, University of Zurich Zurich Switzerland; ^7^ Department of General Internal Medicine Inselspital, Bern University Hospital, University of Bern Bern Switzerland; ^8^ Institute of Primary Health Care (BIHAM), University of Bern Bern Switzerland; ^9^ Department of Cardiology Lausanne University Hospitals Lausanne Switzerland

**Keywords:** acute coronary syndrome, education, health care access, secondary cardiovascular prevention

## Abstract

**Background:**

Despite universal coverage, inequities persist in acute coronary syndrome (ACS) care. This study examines how educational levels impact the quality and outcomes of health care.

**Methods:**

A cohort of ACS patients hospitalized in five Swiss university hospitals was categorized into four educational levels (EL) with EL1 defined as lower than vocational school and EL4 as a university degree. The use of medical therapies, achievement of preventive targets and risk of clinical events were evaluated across ELs at baseline (*N* = 6040), 1‐year (*N* = 5756) and 5‐years (*N* = 2253) and presented with adjusted marginal odds ratios (mOR), average marginal effect (AME) and hazard ratios (HRs).

**Results:**

Among 6040 patients, the mean age was 63 years, and 81% were male. Participants with lower EL had a greater burden of cardiovascular risk factors at baseline. Compared with EL4 participants EL1 participants had lower adherence to cardiac rehabilitation (mOR = .6 [95% CI .5–.8], AME = −10%) and were less likely to be followed by a cardiologist (mOR .6 [95% CI .5–.8], AME = −6%). Use of medical therapies did neither differ across EL at discharge nor during follow‐up. At 1 year, smoking cessation (mOR = .7 [95% CI .5–.9], AME = −10%) and weight reduction ≥5% among overweight or obese participants (mOR = .7 [95% CI .5–.9], AME = −6%) were less frequent in individuals with EL1 compared with EL4. At long term, achievement of LDL‐C <1.8 mmol/L (<70 mg/dL) (mOR = .6 [95% CI .4–.9], AME = −9%) was less frequent in individuals with EL1 compared with EL4. Lower EL was associated with an increased risk of major acute coronary event (MACE) at short‐ (aHR = 1.4 [95% CI 1.0–2.0] for EL1 vs. EL4) and long term (aHR = 1.3 [95% CI 1.0–1.6] for EL1 vs. EL4) and all‐cause death at long term (aHR = 1.6 [95% CI 1.1–2.2] for EL1 vs. EL4).

**Conclusion:**

In Switzerland, disparities in ACS care and outcomes remain across EL, emphasising the need for tailored interventions to reduce inequities.

## Graphical abstract

Despite universal healthcare in Switzerland, disparities in post‐acute coronary syndrome (ACS) care persist across educational levels. In a cohort of 6040 patients, those with the lowest education had more cardiovascular risk factors and were less likely to attend cardiac rehabilitation or receive cardiology follow‐up. Preventive targets like smoking cessation, weight loss and LDL‐C control were less often achieved. Although medication use was similar, lower education was linked to higher mortality. These findings highlight the need for targeted strategies to address educational inequities in ACS care.

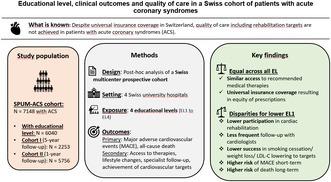

## 1 INTRODUCTION

The association between low socioeconomic status (SES) and higher risk of cardiovascular diseases (CVD) has been described in large populational studies.^1^, ^2^, ^3^, ^4^ Although SES is not integrated as an independent variable in the risk estimation per se, the 2021 ESC prevention guidelines consider low SES as a risk modifier.^5^ Several determinants can be used as a proxy for SES, such as income, education level (EL), occupation, social network and wealth. Among the measurable determinants of SES, EL has been validated as a social determinant of cardiovascular health.^6^, ^7^, ^8^ Recently, the EUROASPIRE IV study gathering 7937 patients from 24 European countries showed that lower EL was associated with worse prognosis after an acute coronary syndrome (ACS) or coronary revascularization and thus independently of other traditional risk factors. Patients with a lower educational level were characterized by a greater tendency to be smokers, overweight or obese, physically inactive, hypertensive and diabetic. However, this study did not integrate the characteristics of the health system per country to potentially disentangle poor access to care versus suboptimal adherence to treatment or healthier lifestyle.^9^

Although Switzerland is considered a country with a high EL in general, some disparities exist in terms of unhealthy lifestyles and cardiovascular risk factors, including a higher prevalence of hypertension and diabetes.^10^ The Swiss healthcare system is characterized by universal health coverage and a compulsory subscription to private health insurance since 1995, which is expected to alleviate disparities related to SES.^11^ Basic healthcare insurance covers guidelines‐recommended treatments after an ACS, including medical therapies, cardiac rehabilitation (CR) and medical follow‐up (FU), and should guarantee equitable access to optimal care, regardless of the level of education. Although covered by basic insurance, there might be residual barriers to accessing the guidelines‐recommended post‐ACS medical therapy and FU, which, combined with lifestyle factors, could influence the achievement of cardiovascular prevention targets and the clinical outcomes of patients with a lower EL.^12^, ^13^, ^14^ With this project, we aimed to study the association between EL and key process and clinical outcomes after ACS, including adherence to medication and preventive targets, healthcare resources utilization and the risk of major adverse cardiovascular events.

## 2 METHODS

### 2.1 Study design, setting and sample

This study is a post hoc analysis of the Special Program University Medicine (SPUM‐ACS) cohort (ClinicalTrials.gov Identifier: NCT01000701 – IRB Protocol 07–131). The study was approved by the local ethics committee (approval number 07–131, CCER), and all participants provided written informed consent. SPUM‐ACS is an investigator‐initiated, multi‐centre prospective cohort study including 7148 enrolled adult individuals (≥18 years) with a main diagnosis of ACS at five Swiss university hospitals (Bern, Geneva, Lausanne, Lugano and Zurich). To plan related secondary analyses, data on educational level were collected prospectively from the beginning of the study. Enrollments spanned from 2007 to 2021. ACS were defined as symptoms compatible with angina pectoris (chest pain, breathlessness) and at least one of the following criteria: (a) ECG ischemic changes, such as persistent or dynamic ST‐segment deviation, T‐wave inversion, new left bundle branch block; (b) evidence of positive conventional or high‐sensitive troponin by local laboratory reference values; (c) known coronary heart disease (CHD) defined by pre‐existing myocardial infarction (MI), coronary artery bypass graft (CABG), percutaneous coronary intervention (PCI), or ≥50% documented stenosis of an epicardial coronary artery in a previous angiography. Exclusion criteria comprised severe physical disability, inability to give consent and less than a year of life expectancy for non‐cardiac reasons. Baseline sociodemographic, lifestyle (e.g., alcohol consumption, smoking), medication use, medical history and clinical data were collected at inclusion in the cohort by a team of study nurses at each participating site. Baseline sociodemographic data were collected at patient inclusion in the cohort, as well as lifestyle habits (e.g., alcohol consumption, smoking), and clinical data, including medication use and medical history, by a team of study nurses at each participating site. Participants underwent FU assessments according to their cohort assignment: participants from cohort I had a blood draw at one‐ and five‐years' FU.

One year after ACS, all participants were invited to attend a face‐to‐face clinical FU visit at the hospital. When this was not possible, a telephone FU visit was set up. If the patient was not reachable, data were collected through a nominated caregiver or the primary care physician. Collected data included adherence to cardiac rehabilitation and medical FU, lifestyle habits, medication use and intermediate cardiovascular events. Participants were asked to bring their treatment list or pillboxes to confirm prescribed medication. They underwent a clinical examination including three sequential blood pressure measurements and weight measurements; fasting blood samples were also obtained to assess low‐density lipoprotein cholesterol (LDL‐C) serum glucose and glycated haemoglobin levels. All cardiovascular events occurring during the FU period were reviewed using prespecified adjudication forms by an adjudication committee comprising three certified expert cardiologists who were masked to patient identities. Following an amendment to the protocol, a second identical FU was performed after 5 years in a subset of the initial population. Not all patients consented to long‐term FU, and not all centres participated in the extended FU phase, resulting in 5756 patients with short‐term FU (cohort II) and 2253 with long‐term FU (cohort I). Short‐term major acute coronary event (MACE) was defined as events occurring within 1 year after the index ACS event. Long‐term MACE referred to events occurring between 1 year and 5 years (Figure S1).

### 2.2 Study exposure and endpoints

Educational level served as study exposure. It was documented for 6040 patients and categorised into four groups at baseline, as per the Swiss Confederation classification^15^: lower than apprenticeship or vocational school (EL1, *N* = 1015), apprenticeship or vocational school (EL2, *N* = 3118), high school graduation (EL3, *N* = 847), university graduation (EL4, *N* = 1060).

The primary clinical endpoints were the incidence of MACE, defined as a composite of cardiovascular death, myocardial infarction, coronary revascularisation and stroke or transient cerebral ischaemic attack.

Secondary endpoints encompassed the documentation of cardiovascular drug therapy prescriptions (aspirin, statins, beta‐blockers, angiotensin‐converting enzyme inhibitors, angiotensin II receptor antagonists) at hospital discharge and FUs; participation in cardiac rehabilitation; adherence to medical FU; adherence to lifestyle measures (i.e., reduction in alcohol consumption if daily alcohol drinker, smoking cessation if smoker); and achievement of secondary cardiovascular prevention targets. Cardiovascular prevention targets were defined as LDL‐C <1.8 mmol/L (<70 mg/dL), systolic blood pressure <140 mmHg, ≥5% reduction in body weight in overweight (BMI 25–29 kg/m^2^) or obese (BMI >30 kg/m^2^) participants, glycated haemoglobin <7% if diabetics, according to the 2016 European guidelines on cardiovascular disease prevention in clinical practice.^16^, ^17^ Systolic blood pressure was calculated as the mean of the second and third measurements at FUs. LDL‐C was determined using Sampson's equation.^18^

### 2.3 Statistical analyses

Baseline characteristics, clinical data and FU data were expressed using mean and standard deviation (SD) or median with interquartile range (IQR) for continuous variables, and frequencies and percentages for categorical variables; and compared across EL using Chi‐squared test, Student's *t*‐test and Kruskal–Wallis test as appropriate. For assessing trends of baseline characteristics, use of medical therapies, medical FU and achievement of secondary prevention targets across EL, the Cochrane–Armitage test was used for categorical variables, and the Jonckheere–Terpstra test was used for continuous variables. To assess the association between ELs and study endpoints, logistic regression models with EL4 as the reference category, unadjusted and adjusted for age and sex (Figure 2), were used. Marginal odds ratios (mOR) and average marginal effects (AME) were obtained with adjusted logistic regression models to provide the average association across the study sample, independent of specific age and sex values. The associations between EL and the hazard of MACE and all‐cause death were assessed using Cox regression models, with EL4 as the reference category. Patients were censored at the first event, death, or last valid contact. Cox regression models were presented unadjusted (model 1), adjusted for age and sex (model 2) and adjusted for age, sex, history of hypertension, history of diabetes, history of myocardial infarction, history of hypercholesterolemia and smoking status at baseline (model 3) (Tables S3–S5). The adjustment set for model 2 was identified using a directed acyclic graph (Figure S2) identifying age and sex as confounders of the relation between the EL and the study endpoints. The adjustment set from model 3 was selected based on previous literature.^19^ All models were evaluated to ensure adherence to their underlying assumptions. A significance level of *p* < .05 was used for all analyses. Statistical analyses were performed using R version 4.3.2 (R Foundation for Statistical Computing, Vienna, Austria).

## 3 RESULTS

### 3.1 Baseline characteristics

Baseline characteristics and clinical data according to EL are presented in Table 1. Significant differences were found where individuals with EL1 tended to be more often female and older compared with the other categories. Participants in the EL1 category were more likely to have a BMI ≥30 kg/m^2^ (25% in the EL1 category vs. 16% in the EL4 category), suffer from diabetes (23% vs. 12%), or hypertension (59% vs. 46%) and to be active smokers (44% vs. 32%). Along with a high prevalence of cardiovascular risk factors in individuals with lower EL, treatments to control those such as aspirin (30% in EL1 vs. 22% in EL4), statins (29% in EL1 vs. 22% in EL4), beta‐blockers (22% in EL1 vs. 16% in EL4) and renin‐angiotensin‐aldosterone system (RAAS) inhibitors (38% in EL1 vs. 27% in EL4) were more common at hospital admission for ACS.

**TABLE 1:** Baseline characteristics according to education level.

	EL1 (*N* = 1015)	EL2 (*N* = 3118)	EL3 (*N* = 847)	EL4 (*N* = 1060)	*p* Value	*p* value for trend
Sociodemographics						
Age	64 (13)	63 (12)	61 (12)	62 (11)	<.001	<.001
Male sex	677/1015 (67%)	2545/3118 (82%)	693/847 (82%)	954/1060 (90%)	<.001	<.001
BMI (kg/m^2^)	27.5 (4.7)	27.2 (4.3)	26.7 (4.2)	26.4 (4.0)	<.001	<.001
Caucasian ethnicity	928/1007 (92%)	3028/3101 (98%)	805/845 (95%)	976/1048 (93%)	<.001	.187
Married or in partnership	649/1015 (64%)	2076/3114 (67%)	564/847 (67%)	735/1058 (69%)	.068	.014
Living alone	289/1014 (29%)	766/3109 (25%)	196/846 (23%)	218/1056 (21%)	<.001	<.001
Working status						
Full time	335/1007 (33%)	1302/3096 (42%)	405/846 (48%)	507/1053 (48%)	<.001	
Part time	65/1007 (6.5%)	247/3096 (8.0%)	80/846 (9.5%)	114/1053 (11%)		
No employment/retired	607/1007 (60%)	1547/3096 (50%)	361/846 (43%)	432/1053 (41%)		
Medical history						
Obesity (BMI ≥30 kg/m^2^)	252/1007 (25%)	691/3078 (22%)	170/835 (20%)	170/1053 (16%)	<.001	<.001
Current smoking	444/1015 (44%)	1278/3105 (41%)	352/846 (42%)	343/1059 (32%)	<.001	<.001
Daily alcohol consumption	266/1000 (27%)	744/3019 (25%)	218/831 (26%)	308/1049 (29%)	.026	.041
Diabetes	231/1015 (23%)	544/3117 (17%)	104/847 (12%)	130/1060 (12%)	<.001	<.001
Hypertension	597/1015 (59%)	1730/3117 (56%)	414/845 (49%)	490/1060 (46%)	<.001	<.001
Myocardial infarction	166/1013 (16%)	384/3114 (12%)	85/845 (10%)	114/1059 (11%)	<.001	<.001
Stroke or transient ischemic attack	39/1014 (3.8%)	112/3117 (3.6%)	38/847 (4.5%)	39/1060 (3.7%)	.681	.817
Peripheral vascular disease	58/1015 (5.7%)	181/3117 (5.8%)	35/846 (4.1%)	35/1060 (3.3%)	.005	.001
Heart failure	23/1014 (2.3%)	33/3117 (1.1%)	8/847 (.9%)	13/1060 (1.2%)	.020	.094
End‐stage kidney disease	5/1014 (.5%)	20/3117 (.6%)	4/847 (.5%)	8/1060 (.8%)	.824	.599
Lung disease (COPD or asthma)	48/1014 (4.7%)	131/3115 (4.2%)	14/846 (1.7%)	16/1056 (1.5%)	<.001	<.001
Liver disease	6/1014 (.6%)	14/3117 (.4%)	6/847 (.7%)	12/1060 (1.1%)	.111	.045
Malignancy	70/1013 (6.9%)	242/3115 (7.8%)	69/846 (8.2%)	79/1060 (7.5%)	.751	.679
Systemic inflammatory disease	31/1014 (3.1%)	83/3117 (2.7%)	16/847 (1.9%)	25/1060 (2.4%)	.419	.196
Lipid profile at baseline						
Total cholesterol	5.0 (4.1 5.8)	4.9 (4.1 5.7)	5.0 (4.2 5.9)	5.0 (4.2 5.9)	<.001	.007
LDL‐C	3.5 (2.8 4.4)	3.6 (2.8 4.4)	3.6 (2.9 4.6)	3.6 (2.9 4.5)	.002	.003
HDL‐C	1.1 (.9 1.4)	1.1 (.9 1.4)	1.1 (.9 1.4)	1.1 (1.0 1.4)	.804	.414
Triglycerides	1.2 (.8 1.8)	1.1 (.7 1.7)	1.1 (.8 1.7)	1.1 (.7 1.7)	<.001	.168
Medication at baseline						
Aspirin	306/1009 (30%)	840/3096 (27%)	183/838 (22%)	237/1057 (22%)	<.001	<.001
Anti‐P2Y12	67/732 (9.2%)	206/2398 (8.6%)	46/691 (6.7%)	51/873 (5.8%)	.021	.003
Statins	289/1006 (29%)	816/3090 (26%)	208/838 (25%)	236/1056 (22%)	.007	<.001
Beta‐blockers	217/1005 (22%)	693/3085 (22%)	141/837 (17%)	168/1055 (16%)	<.001	<.001
Renin‐angiotensin system inhibitors	382/1006 (38%)	1048/3082 (34%)	251/834 (30%)	285/1053 (27%)	<.001	<.001
Oral antidiabetics (if diabetics)	151/229 (66%)	355/535 (66%)	69/103 (67%)	92/128 (72%)	.661	.272
Insulin (if diabetics)	59/229 (26%)	155/535 (29%)	19/103 (18%)	35/129 (27%)	.167	.666
Clinical data						
Type of ACS						
NSTEMI	453/1015 (45%)	1293/3118 (41%)	336/847 (40%)	426/1060 (40%)	.022	
STEMI	508/1015 (50%)	1693/3118 (54%)	485/847 (57%)	592/1060 (56%)		
Unstable angina	54/1015 (5.3%)	129/3118 (4.1%)	25/847 (3.0%)	39/1060 (3.7%)		
Undertermined	0/1015 (0%)	3/3118 (<.1%)	1/847 (.1%)	3/1060 (.3%)		
Kilipp class II–IV	133/962 (14%)	328/3014 (11%)	90/799 (11%)	91/1018 (8.9%)	.007	.003
LVEF						
LVEF >40%	722/894 (81%)	2285/2806 (81%)	628/775 (81%)	828/970 (85%)	.024	.010
LVEF ≤40%	172/894 (19%)	521/2806 (19%)	147/775 (19%)	142/970 (15%)		
Discharge destination						
Home	594/1014 (59%)	1513/3107 (49%)	545/845 (64%)	738/1058 (70%)	<.001	
Other hospital	292/1014 (29%)	1299/3107 (42%)	209/845 (25%)	208/1058 (20%)		
Rehabilitation center	128/1014 (13%)	295/3107 (9.5%)	91/845 (11%)	112/1058 (11%)		

*Note*: Categorical variables are expressed as percentages (count with percentage), while continuous variables are reported as either mean ± standard deviation or median with interquartile range. BMI was missing for 67 participants, total cholesterol for 302, LDL‐C for 405, HDL‐C for 392 and triglycerides for 376. *p* values were determined using Chi‐squared tests for categorical variables, Student's *t*‐test for normally distributed continuous variables, and Kruskal–Wallis test for non‐normally distributed continuous variables. For assessing trends, the Cochrane–Armitage test was used for categorical variables, and the Jonckheere–Terpstra test was employed for continuous variables.

Abbreviations: BMI, body mass index; COPD, chronic obstructive pulmonary disease; EL, education level; HDL‐C, high‐density lipoprotein cholesterol; LDL‐C, low‐density lipoprotein cholesterol; LVEF, left ventricular ejection fraction.

### 3.2 Adherence to cardiac rehabilitation and medical follow‐up

Adherence to cardiac rehabilitation and medical FU during the first year post‐ACS are summarized in Table 2. The data show significant disparities in the post‐ACS care and FU depending on EL: compared with adults with EL4, adults with EL1 had lower adherence to cardiac rehabilitation (e.g., 67% in EL1 vs. 78% in EL4, *p* for trend <.001), including after accounting for age and sex (e.g., mOR .62, 95% CI .50–.75, AME = −9.5% in EL1 vs. EL4).

**TABLE 2:** Attendance to cardiac rehabilitation and medical follow‐up during the first year post ACS according to education levels.

	EL1 (*N* = 1015)	EL2 (*N* = 3118)	EL3 (*N* = 847)	EL4 (*N* = 1060)	*p*‐Value	*p* for trend	EL1 vs. EL4		EL2 vs. EL4		EL3 vs. EL4	
	*n*/*N* (%)	*n*/*N* (%)	*n*/*N* (%)	*n*/*N* (%)			Marginal OR (95% CI) [AME (95% CI)]	*p*‐Value	Marginal OR (95% CI) [AME (95% CI)]	*p*‐Value	Marginal OR (95% CI) [AME (95% CI)]	*p*‐Value
Attendance to cardiac rehabilitation	625/929 (67%)	2165/2917 (74%)	605/788 (77%)	779/1000 (78%)	<.001	<.001	.62 (.50, .75) [−9.5 (−13.4, −5.5)]	<.001	.83 (.70, .98) [−3.3 (−6.3, −.4)]	.031	.90 (.72, 1.11) [−2.0 (−5.9, 1.9)]	.318
Any medical follow‐up	891/903 (99%)	2830/2872 (99%)	744/763 (98%)	963/972 (99%)	.058	.829	.67 (.28, 1.62) [−.4 (−1.4, .5)]	.375	.62 (.30, 1.28) [−.6 (−1.3, .2)]	.198	.37 (.17, .83) [−1.5 (−2.7, −.3)]	.016
Medical follow‐up with a general practitioner	826/878 (94%)	2617/2810 (93%)	676/737 (92%)	834/957 (87%)	<.001	<.001	2.10 (1.50, 2.96) [6.1 (3.4, 8.8)]	<.001	1.92 (1.51, 2.44) [5.5 (3.3, 7.8)]	<.001	1.62 (1.17, 2.23) [4.4 (1.5, 7.2)]	.003
Medical follow‐up with a cardiologist	713/875 (81%)	2252/2812 (80%)	659/739 (89%)	847/961 (88%)	<.001	<.001	.64 (.49, .83) [−5.7 (−8.9, −2.4)]	.001	.56 (.45, .69) [−7.7 (−10.3, −5.2)]	<.001	1.09 (.81, 1.48) [.9 (−2.2, 4.0)]	.565

*Note*: Data are expressed as counts with percentages. *p* values were determined using Chi‐squared tests for categorical variables. For assessing trends, the Cochrane‐Armitage test was used for categorical variables, and the Jonckheere‐Terpstra test was employed for continuous variables. Marginal odds ratios and AME with 95% confidence intervals obtained with logistic regression models accounting for age and sex are presented.

Abbreviations: AME, average marginal effect; CI, confidence interval; OR, odds ratio.

Adherence to medical FUs was high across all EL (98%), however, patients with lower EL were more frequently followed up by a general practitioner (94% in EL1 vs. 87% in EL4, *p* for trend <.001), while patients with higher EL were more often followed up by a cardiologist (e.g., 81% in EL1 versus 88% in EL4, *p* for trend <.001). This pattern persisted after accounting for age and sex (mOR for FU by general practitioner 2.10, 95% CI 1.50–2.96 in EL1 vs. EL4, AME = +6.5%; and by cardiologist .64, 95% CI .49–.83 in EL1 vs. EL4, AME = −5.7%).

### 3.3 Adherence to secondary cardiovascular prevention

Cardiovascular treatment prescriptions at discharge are summarized in Table 3. At discharge for ACS, the majority of patients received guideline‐recommended medical therapies, with no pattern associated with EL (*p* for trend >.05), including after accounting for age and sex: 99% received aspirin, 98% anti‐P2Y12, 98% statins, 89% renin‐angiotensin system inhibitors and 80% beta‐blockers.

**TABLE 3:** Prescription of recommended cardiovascular therapies at discharge and one‐year and long‐term follow‐ups according to education level.

	EL1 (*N* = 1015)	EL2 (*N* = 3118)	EL3 (*N* = 847)	EL4 (*N* = 1060)	*p‐*Value	*p* for trend	EL1 vs. EL4		EL2 vs. EL4		EL3 vs. EL4	
	*n*/*N* (%)	*n*/*N* (%)	*n*/*N* (%)	*n*/*N* (%)			Marginal OR (95% CI) [AME (95% CI)]	*p*‐Value	Marginal OR (95% CI) [AME (95% CI)]	*p*‐Value	Marginal OR (95% CI) [AME (95% CI)]	*p*‐Value
Discharge												
Aspirin	1011/1015 (100%)	3094/3117 (99%)	840/847 (99%)	1050/1060 (99%)	.501	.163	3.16 (.96, 10.40) [.7 (−.0, 1.5)]	.058	1.43 (.67, 3.01) [.3 (−.4, 1.0)]	.353	1.16 (.44, 3.07) [.1 (−.8, 1.1)]	.762
Anti‐P2Y12	969/983 (99%)	2977/3036 (98%)	819/832 (98%)	1021/1040 (98%)	.703	.787	1.54 (.76, 3.14) [.7 (−.4, 1.8)]	.231	1.01 (.60, 1.70) [.0 (−1.0, 1.0)]	.982	1.21 (.59, 2.47) [.3 (−.9, 1.6)]	.601
Statins	987/1014 (97%)	3060/3117 (98%)	828/847 (98%)	1038/1060 (98%)	.427	.674	.88 (.49, 1.58) [−.3 (−1.6, 1.0)]	.667	1.19 (.73, 1.96) [.3 (−.7, 1.4)]	.486	.92 (.49, 1.71) [−.2 (−1.6, 1.2)]	.794
ACEi/ARB	909/1014 (90%)	2755/3117 (88%)	762/847 (90%)	936/1060 (88%)	.443	.689	1.21 (.91, 1.60) [1.9 (−.9, 4.6)]	.183	1.03 (.83, 1.28) [.3 (−2.0, 2.6)]	.804	1.21 (.90, 1.62) [1.9 (−1.0, 4.7)]	.197
Beta‐blockers	834/1014 (82%)	2448/3116 (79%)	688/847 (81%)	855/1060 (81%)	.041	.917	1.12 (.89, 1.40) [1.6 (−1.7, 5.0)]	.342	.88 (.74, 1.05) [−2.1 (−4.9, .7)]	.153	1.03 (.82, 1.29) [.4 (−3.1, 4.0)]	.814
Oral antidiabetics (if diabetic)	146/228 (64%)	375/542 (69%)	69/104 (66%)	96/130 (74%)	.246	.100	.70 (.43, 1.14) [−7.6 (−17.7, 2.6)]	.152	.83 (.54, 1.27) [−3.9 (−12.6, 4.7)]	.388	.72 (.41, 1.26) [−7.0 (−19.0, 4.9)]	.247
Insulin (if diabetic)	78/229 (34%)	204/543 (38%)	36/104 (35%)	51/130 (39%)	.702	.452	.80 (.50, 1.26) [−5.3 (−15.9, 5.4)]	.330	.93 (.63, 1.38) [−1.7 (−11.0, 7.7)]	.728	.82 (.48, 1.40) [−4.6 (−17.1, 7.8)]	.466
First follow‐up (1 year)												
Aspirin	917/953 (96%)	2843/2957 (96%)	763/797 (96%)	978/1012 (97%)	.794	.706	.92 (.57, 1.50) [−.3 (−1.9, 1.4)]	.738	.88 (.60, 1.30) [−.4 (−1.7, .9)]	.525	.74 (.45, 1.19) [−1.2 (−3.0, .7)]	.213
Anti‐P2Y12	710/873 (81%)	2341/2804 (83%)	584/741 (79%)	751/948 (79%)	.003	.014	1.20 (.94, 1.51) [2.8 (−.9, 6.5)]	.137	1.35 (1.12, 1.62) [4.5 (1.6, 7.5)]	.002	.97 (.77, 1.23) [−.5 (−4.5, 3.5)]	.803
Statins	875/953 (92%)	2758/2955 (93%)	728/797 (91%)	933/1011 (92%)	.160	.624	1.09 (.78, 1.52) [.6 (−1.8, 3.0)]	.614	1.24 (.94, 1.63) [1.5 (−.5, 3.4)]	.122	.92 (.66, 1.29) [−.7 (−3.3, 2.0)]	.621
ACEi/ARB	775/953 (81%)	2367/2954 (80%)	629/797 (79%)	774/1012 (76%)	.037	.004	1.42 (1.14, 1.77) [5.8 (2.1, 9.4)]	.002	1.27 (1.07, 1.51) [4.1 (1.0, 7.1)]	.007	1.19 (.95, 1.49) [3.0 (−.9, 6.9)]	.132
Beta‐blockers	732/953 (77%)	2243/2955 (76%)	574/797 (72%)	711/1012 (70%)	<.001	<.001	1.38 (1.12, 1.70) [6.3 (2.3, 10.2)]	.002	1.33 (1.13, 1.56) [5.5 (2.3, 8.8)]	.001	1.08 (.88, 1.33) [1.6 (−2.6, 5.8)]	.447
Oral antidiabetics (if diabetic)	151/212 (71%)	380/508 (75%)	72/92 (78%)	86/119 (72%)	.558	.640	1.02 (.61, 1.71) [.5 (−9.9, 10.9)]	.926	1.19 (.76, 1.87) [3.5 (−5.5, 12.5)]	.437	1.41 (.75, 2.67) [6.5 (−5.2, 18.3)]	.284
Insulin (if diabetic)	68/212 (32%)	174/510 (34%)	26/92 (28%)	36/119 (30%)	.648	.515	1.11 (.68, 1.83) [2.3 (−8.3, 12.9)]	.676	1.21 (.79, 1.87) [4.2 (−5.0, 13.4)]	.380	.91 (.50, 1.66) [−1.9 (−14.2, 10.4)]	.765
Second follow‐up (6.6 years)												
Aspirin	345/384 (90%)	921/1013 (91%)	306/343 (89%)	434/482 (90%)	.794	.762	1.05 (.66, 1.69) [.5 (−3.6, 4.5)]	.824	1.09 (.75, 1.60) [.8 (−2.5, 4.1)]	.643	.82 (.52, 1.31) [−1.9 (−6.3, 2.6)]	.410
Anti‐P2Y12	39/383 (10%)	121/1013 (12%)	41/343 (12%)	55/481 (11%)	.821	.709	1.00 (.64, 1.56) [−.0 (−4.4, 4.4)]	.998	1.10 (.77, 1.55) [.9 (−2.6, 4.4)]	.609	1.10 (.71, 1.72) [1.0 (−3.5, 5.5)]	.659
Statins	319/383 (83%)	897/1013 (89%)	286/343 (83%)	401/482 (83%)	.006	.174	1.20 (.82, 1.74) [2.5 (−2.7, 7.7)]	.350	1.67 (1.22, 2.29) [6.3 (2.2, 10.5)]	.001	1.02 (.70, 1.49) [.3 (−5.2, 5.8)]	.922
ACEi/ARB	282/383 (74%)	751/1012 (74%)	230/343 (67%)	327/482 (68%)	.012	.005	1.44 (1.05, 1.96) [7.4 (1.1, 13.7)]	.022	1.40 (1.10, 1.78) [6.9 (1.8, 12.1)]	.007	1.05 (.78, 1.42) [1.1 (−5.5, 7.7)]	.743
Beta‐blockers	248/383 (65%)	638/1013 (63%)	179/343 (52%)	270/482 (56%)	<.001	<.001	1.33 (1.00, 1.76) [6.8 (.0, 13.5)]	.050	1.27 (1.02, 1.59) [5.8 (.4, 11.1)]	.034	.83 (.63, 1.11) [−4.5 (−11.5, 2.5)]	.207
Oral antidiabetics (if diabetic)	49/65 (75%)	118/153 (77%)	31/43 (72%)	41/53 (77%)	.912	.993	.81 (.33, 1.99) [−3.9 (−20.1, 12.3)]	.639	.90 (.42, 1.96) [−1.8 (−15.1, 11.5)]	.793	.71 (.27, 1.85) [−6.4 (−24.2, 11.5)]	.485
Insulin (if diabetic)	21/65 (32%)	57/153 (37%)	15/43 (35%)	22/53 (42%)	.765	.384	.70 (.31, 1.58) [−8.5 (−27.6, 10.7)]	.386	.83 (.42, 1.66) [−4.3 (−21.0, 12.3)]	.605	.86 (.37, 2.02) [−3.6 (−23.9, 16.8)]	.732

*Note*: Data are expressed as count with percentage. *p* values were determined using Chi‐squared tests for categorical variables. For assessing trends, the Cochrane–Armitage test was used for categorical variables, and the Jonckheere–Terpstra test was employed for continuous variables. Marginal odds ratios and AME with 95% confidence interval obtained with logistic regression models accounting for age and sex are presented.

Abbreviations: ACEi, angiotensin‐converting enzyme inhibitor; AME, average marginal effect; ARB, angiotensin receptor blockers; CI, confidence interval; OR, odds ratio.

At the one‐year FU, adherence to long‐term guidelines recommended cardiovascular therapies remained robust across all EL groups, with 96% receiving aspirin, 82% anti‐P2Y12 and 93% statins. However, individuals with lower EL were more likely to receive angiotensin‐converting enzyme inhibitors (ACEi) or angiotensin receptor blockers (ARB, e.g., 81% in EL1 vs. 76% in EL4, *p* for trend = .004) and beta‐blockers (e.g., 77% in EL1 vs. 70% in EL4, *p* for trend <.001). These patterns persisted after accounting for age and sex: individuals with EL1 and EL2 respectively had 42% (95% CI 14%–77%, AME = 5.8%) and 27% (95% CI 7%–51%, AME = 4.1%) greater odds of receiving ACEi or ARB; and respectively 38% (95% CI 12%–70%, AME = 6.3%) and 33% (95% CI 13%–56%, AME = 5.5%) greater odds of receiving beta‐blockers compared with the EL4 group.

At the second FU, extending beyond 5 years post‐ACS, the prescription rate of anti‐P2Y12 dropped to 12%, with no education‐related pattern. Among individuals with lower EL, a persisting pattern toward greater use of ACEi or ARB and beta‐blockers was observed.

### 3.4 Achievement of secondary prevention targets

Regarding achievement of secondary prevention targets and lifestyle modifications (Table 4), individuals with lower EL were less likely than those with higher EL to lose ≥5% body weight from baseline if overweight or obese (e.g., 18% in EL1 vs. 22% in EL4, *p* for trend = .014; mOR .67, 95% CI .50–.90, AME = −6.3%) and stop smoking (e.g., 45% in EL1 vs. 55% in EL4, *p* for trend = .006; mOR .67, 95% CI .50–.90, AME = −9.9%) 1 year after ACS. There was no association between EL and achievement of LDL‐C <1.8 mmol/L (<70 mg/dL) and systolic blood pressure <140 mmHg one year after ACS. At the second FU, extending beyond 5 years post‐ACS, a gap emerges regarding achieved cholesterol target: individuals with lower EL less frequently reached or maintained the LDL‐C target of <1.8 mmol/L (<70 mg/dL) compared with individuals with the highest EL (e.g., 18% in EL1 vs. 27% in EL4, *p* for trend = .009; mOR .61, 95% CI .41–.89, AME = −8.6%).

**TABLE 4:** Achievement of secondary prevention targets at one‐year and five‐year follow‐ups according to education levels.

	EL1 (*N* = 1015)	EL2 (*N* = 3121)	EL3 (*N* = 847)	EL4 (*N* = 1060)	*p*‐Value	*p* for trend	EL1 vs. EL4		EL2 vs. EL4		EL3 vs. EL4	
	*n*/*N* (%)	*n*/*N* (%)	*n*/*N* (%)	*n*/*N* (%)			Marginal OR (95% CI) [AME (95% CI)]	*p*‐Value	Marginal OR (95% CI) [AME (95% CI)]	*p*‐Value	Marginal OR (95% CI) [AME (95% CI)]	*p*‐Value
First follow‐up (1 year)												
LDL‐C <1.8 mmol/l	115/549 (21%)	277/1342 (21%)	103/477 (22%)	146/615 (24%)	.472	.169	.89 (.67, 1.18) [−2.0 (−6.8, 2.9)]	.426	.86 (.68, 1.08) [−2.7 (−6.6, 1.3)]	.181	.92 (.69, 1.23) [−1.4 (−6.4, 3.6)]	.587
HbA1c <7% (if diabetic)	41/74 (55%)	73/141 (52%)	28/38 (74%)	37/59 (63%)	.080	.118	.64 (.31, 1.31) [−10.8 (−27.9, 6.3)]	.221	.60 (.32, 1.12) [−12.3 (−26.9, 2.3)]	.108	1.68 (.69, 4.06) [10.9 (−7.2, 28.9)]	.253
Systolic blood pressure <140 mmHg	433/636 (68%)	1659/2217 (75%)	418/585 (71%)	549/755 (73%)	.006	.493	.82 (.65, 1.03) [−4.1 (−8.9, .6)]	.086	1.11 (.92, 1.33) [2.0 (−1.6, 5.5)]	.266	.88 (.70, 1.12) [−2.5 (−7.3, 2.2)]	.295
Weight reduction ≥5% (if overweight or obese)	101/573 (18%)	362/1805 (20%)	109/437 (25%)	121/543 (22%)	.025	.014	.67 (.50, .90) [−6.3 (−11.1, −1.6)]	.009	.84 (.67, 1.06) [−2.9 (−6.9, 1.1)]	.150	1.14 (.85, 1.53) [2.4 (−3.0, 7.8)]	.384
Smoking cessation (if smoker)	180/403 (45%)	596/1195 (50%)	163/322 (51%)	181/327 (55%)	.039	.006	.67 (.50, .90) [−9.9 (−17.2, −2.6)]	.009	.81 (.64, 1.04) [−5.2 (−11.3, .9)]	.098	.83 (.61, 1.14) [−4.5 (−12.2, 3.2)]	.252
Alcohol consumption reduction (if daily alcohol consumer)	80/219 (37%)	243/661 (37%)	70/197 (36%)	81/271 (30%)	.237	.073	1.23 (.84, 1.79) [4.5 (−3.9, 12.9)]	.292	1.31 (.97, 1.77) [6.0 (−.6, 12.6)]	.079	1.20 (.81, 1.77) [4.0 (−4.6, 12.6)]	.360
Second follow‐up (6.6 years)												
LDL‐C <1.8 mmol/l	52/295 (18%)	168/768 (22%)	54/278 (19%)	101/371 (27%)	.016	.009	.61 (.41, .89) [−8.6 (−14.9, −2.2)]	.010	.78 (.58, 1.03) [−4.6 (−10.0, .7)]	.083	.68 (.47, 1.00) [−6.7 (−13.2, −.2)]	.048
HbA1c <7% (if diabetic)	19/42 (45%)	34/96 (35%)	15/32 (47%)	18/43 (42%)	.574	.856	.93 (.38, 2.25) [−1.8 (−23.5, 19.8)]	.870	.69 (.33, 1.45) [−8.7 (−26.4, 9.0)]	.330	1.22 (.49, 3.05) [5.0 (−17.7, 27.7)]	.667
Systolic blood pressure <140 mmHg	101/159 (64%)	300/429 (70%)	95/152 (63%)	141/206 (68%)	.251	.821	.81 (.52, 1.26) [−4.7 (−14.5, 5.2)]	.350	1.06 (.74, 1.51) [1.2 (−6.3, 8.8)]	.747	.72 (.47, 1.12) [−7.4 (−17.3, 2.5)]	.142
Weight reduction ≥5% (if overweight or obese)	43/166 (26%)	126/484 (26%)	28/130 (22%)	35/174 (20%)	.359	.103	1.09 (.65, 1.81) [1.4 (−7.4, 10.3)]	.753	1.27 (.84, 1.92) [4.4 (−2.9, 11.6)]	.252	1.10 (.64, 1.89) [1.6 (−7.8, 11.1)]	.736
Smoking cessation (if smoker)	81/166 (49%)	211/430 (49%)	58/142 (41%)	87/161 (54%)	.147	.674	.81 (.52, 1.26) [−5.2 (−16.1, 5.7)]	.353	.82 (.57, 1.18) [−4.9 (−13.9, 4.2)]	.293	.59 (.37, .93) [−13.1 (−24.3, −2.0)]	.023
Alcohol consumption reduction (if daily alcohol consumer)	57/118 (48%)	121/287 (42%)	30/84 (36%)	52/153 (34%)	.078	.011	1.66 (1.01, 2.73) [12.2 (.4, 24.1)]	.045	1.39 (.93, 2.09) [7.8 (−1.6, 17.2)]	.110	1.01 (.58, 1.75) [.2 (−12.4, 12.7)]	.980

*Note*: Data are expressed as counts with percentages. *p* values were determined using Chi‐squared tests for categorical variables. For assessing trends, the Cochrane‐Armitage test was used for categorical variables, and the Jonckheere‐Terpstra test was employed for continuous variables. Marginal odds ratios and AME with 95% confidence intervals obtained with logistic regression models accounting for age and sex are presented.

Abbreviations: AME, average marginal effect; CI, confidence interval; OR, odds ratio; LDL‐C, low density lipoprotein cholesterol.

### 3.5 Risk of major adverse cardiovascular events

**FIGURE 1:**
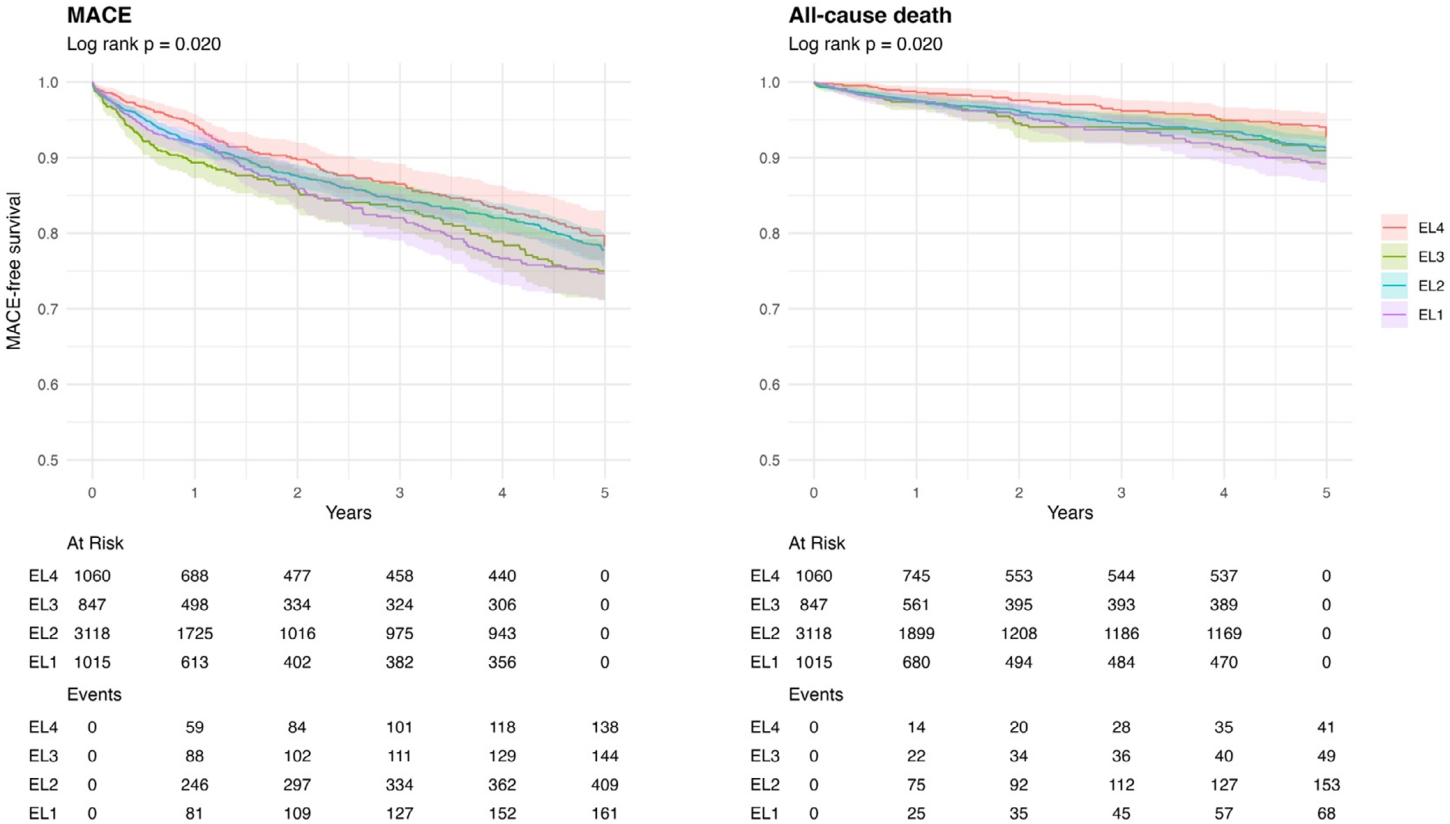
Kaplan Meier curves for MACE and all‐cause death according to education levels. EL, education level; MACE, major adverse cardiovascular event.

**FIGURE 2:**
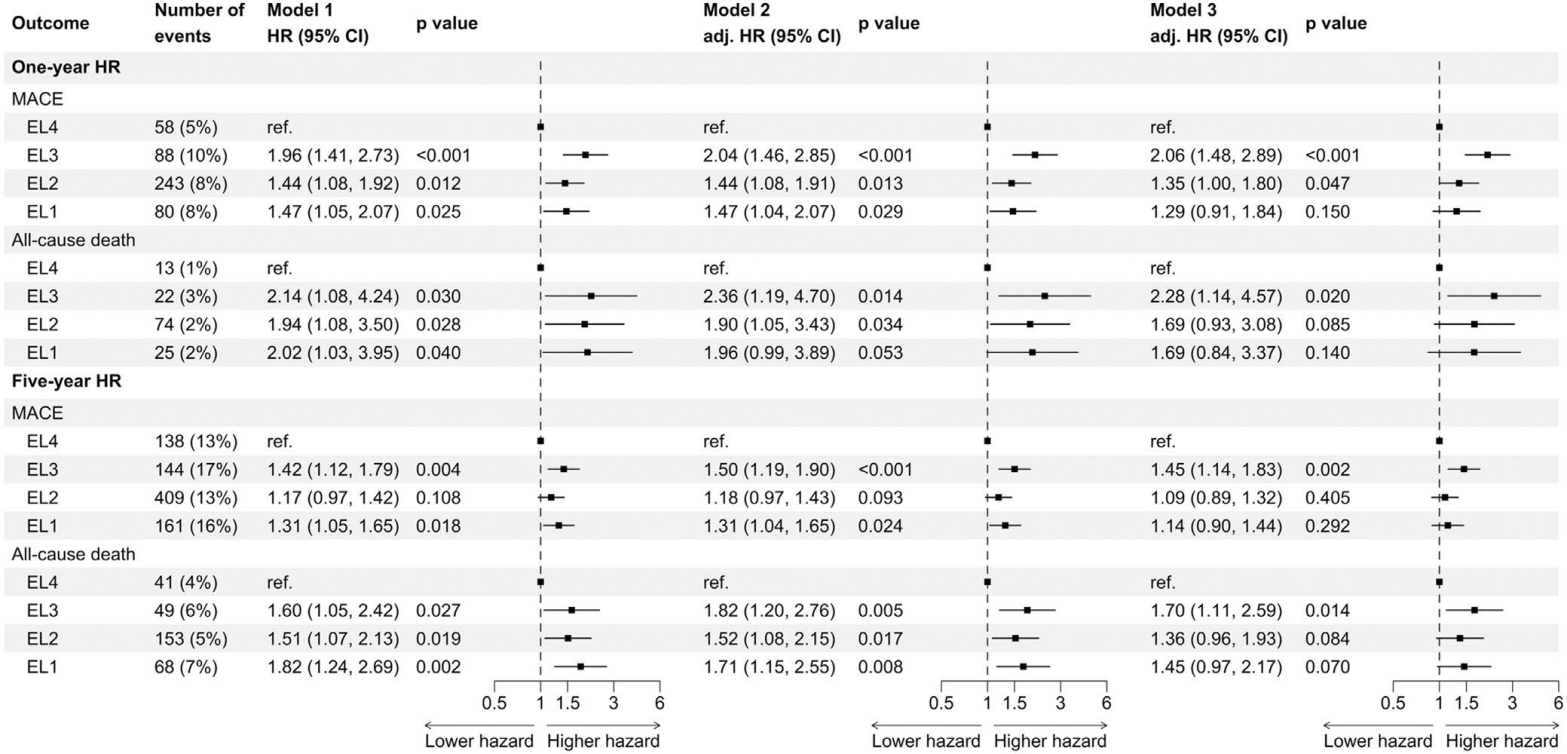
Association of education levels with the risk of major adverse events. Data are presented as count and percentage. Hazard ratios with 95% confidence interval are presented unadjusted (model 1), adjusted for age and sex (model 2), and for age, sex, BMI, history of hypertension, smoking status, diabetes, hypercholesterolemia, and previous MI (model 3). EL, education level; MACE, major adverse cardiovascular event.

A total of 311 patients died, and 852 experienced a recurrent MACE during the FU periods. The cohort used for the 1‐year FU served as the base cohort, and a reduced cohort was available for extended FU. Long‐term survival and survival‐free MACE curves stratified according to EL are presented in Figure 1. After accounting for key confounders such as age and sex, lower EL was associated with short‐term risk of MACE (Figure 2, One‐year HR, aHR = 1.47, 95% CI 1.04–2.07 for EL1; 1.44, 1.08–1.91 for EL2; and 2.04, 1.46–2.85 for EL3 compared with EL4). This association persisted over the 5‐year follow‐up, with adjusted hazard ratios remaining elevated among individuals with lower EL compared with those with tertiary education (Figure 2, Five‐year HR, Model 2, aHR = 1.31, 95% CI 1.04–1.65 for EL1; 1.18, .97–1.43 for EL2; and 1.50, 1.19–1.90 for EL3 compared with EL4). Similarly, the incidence of all‐cause death differed significantly across EL categories (log‐rank *p* = .005), with less‐educated individuals consistently exhibiting a higher risk of death compared with their more‐educated counterparts (Figure 2, Five‐year HR, Model 2, aHR = 1.71, 95% CI 1.15–2.55 for EL1; 1.52, 1.08–2.15 for EL2; and 1.82, 1.20–2.76 for EL3 compared with EL4).

## 4 DISCUSSION

### 4.1 Impact of educational level on secondary prevention

The study reveals EL as a contributing factor to disparities in cardiac rehabilitation adherence, medical FU and the risk of long‐term major adverse events after ACS. Notably, participants with lower EL were less likely to follow a cardiac rehabilitation programme. They were also more inclined to consult GPs, while those with higher EL were more likely to seek care from cardiologists. Additionally, participants with lower EL were at increased risk of all‐cause death and recurrent MACE over the long term.

Similar disparities in cardiovascular outcomes according to EL have been reported in other European countries. In Germany, a recent study aiming at exploring the link between cumulative social disadvantage and CVD burden and mortality within a broad segment of the general German population^20^ highlighted that individuals with prevalent CVD typically had a lower SES, characterised by diminished education, occupation and household net‐income scores. In France, data from the CONSTANCES cohorts showed that individuals with lower ELs had a higher prevalence of cardiovascular risk factors and lower participation in prevention programmes, despite universal health coverage.^21^ In Spain, a recent study highlighted worse cardiovascular outcomes in individuals with lower socioeconomic status, including education, particularly in regions with less structured secondary prevention.^22^ In Italy, the Moli‐sani study demonstrated that lower education was associated with higher cardiovascular risk and mortality, partly mediated by lifestyle and behavioural factors.^23^ In the United Kingdom, data from the UK Biobank and the MINAP registry revealed persistent educational disparities in cardiac rehabilitation uptake and lifestyle modification, despite equitable access to prescriptions under the NHS.^24^ These findings corroborate previous research^25^, ^26^, ^27^ and highlight the critical need to target prevention of cardiovascular risk factors and access to appropriate medical FU to mitigate health inequities by educational status in the context of ACS.

### 4.2 Clinical evidence and long‐term follow‐up

Taken together, these observations underscore a broader concern and gain additional weight when examined alongside recent clinical evidence on long‐term cardiovascular outcomes. A recent meta‐analysis of 19 randomized controlled trials comparing PCI with optimal medical therapy (OMT) in patients with chronic coronary syndromes (CCS) demonstrated a significant reduction in cardiovascular mortality and angina burden in the PCI group, particularly after 3 years of FU and in younger populations.^28^ While our study focused on ACS, these findings highlight the importance of long‐term continuity of care following revascularization. Socioeconomic disparities in access to secondary prevention—such as cardiac rehabilitation and specialist FU—may blunt the potential benefits observed in clinical trials, underscoring the need for tailored care strategies that ensure sustained engagement, particularly among patients with lower educational status.

### 4.3 Uniform prescribing, unequal outcomes

Our results also showed that prescription rates of recommended medical therapies were overall uniform across EL, suggesting that the Swiss healthcare system is efficient in levelling inequities at the prescription level. This standardized approach underscores the commitment to evidence‐based medicine, where healthcare professionals prioritize the consistent application of proven therapies, irrespective of socioeconomic factors and the system's efficiency in delivering optimal patient outcomes and minimizing disparities based on educational attainment, and aligns with previous findings.^29^ However, prognostic and achievement of cardiovascular prevention targets were worst for participants in the lowest education strata. This association may be partly explained by the well‐established link between lower education and reduced medication adherence, likely driven by differences in health literacy, self‐management capacity and structural or psychosocial barriers.^30^

In both unadjusted and age‐ and sex‐adjusted models, individuals in lower educational strata were at greater risk of all‐cause death and of experiencing a short‐term recurrent MACE during the study FU, but this relationship attenuated after accounting for age, sex, cardiovascular risk factors (model 3), which may suggest that cardiovascular risk factors such as obesity, smoking and hypertension mediate the association between EL and adverse clinical outcomes (i.e. these risk factors, influenced by education, affect lifestyle choices and subsequently the risk of adverse events). Adjusting for cardiovascular risk factors, the potential mediators, accounts for their influence (i.e. controlling for the indirect effect of EL on the risk of adverse events through cardiovascular risk factors and lifestyle), which could explain the reduced strength of the observed associations.

### 4.4 Multifactorial barriers to optimal care post‐ACS

The observation from our cohort that lower EL is associated with lower adherence to cardiac rehabilitation and being followed up by a GP rather than a cardiologist can be explained and justified through several lenses. First, SES shapes cardiovascular risk from early childhood through factors such as unhealthy diets, living in polluted or noisy environments and limited access to safe infrastructure for physical activity.^31^, ^32^, ^33^ Second, access to specialised healthcare is significantly influenced by financial barriers and health insurance coverage,^34^ both of which are closely tied to educational attainment. Individuals with lower educational levels generally have lower incomes, making specialised care less affordable. GPs, being more accessible and less costly, often become the primary point of care. Additionally, the quality and extent of health insurance, which tend to vary with economic status and correlate with EL, can further influence access to specialists, with individuals holding more comprehensive coverage being more likely to receive specialised care.^35^ Moreover, GPs themselves may face financial constraints that limit their ability to prescribe newer or more effective medications, which are often more expensive, thereby reducing access to optimal pharmacological therapies. Third, education plays a crucial role in health literacy, which encompasses an individual's ability to obtain, process and understand basic health information for making informed health decisions, as well as treatment adherence. Individuals with higher educational levels are often more aware of their health needs and can navigate through the complexities of the healthcare system, enabling them to seek specialist care more efficiently.^36^ Fourth, socioeconomic status and social determinants, including networks and social support, also impact healthcare utilisation.^37^ Individuals with higher socioeconomic status often have access to networks that can recommend and facilitate consultations with specialists, a luxury not always available to those with lower socioeconomic status, who might also reside in areas with limited healthcare resources, including access to specialists. Perceptions around the necessity of and trust in healthcare providers can also differ across educational levels. Lastly, education affects perceptions of illness severity and the need for specialised care, with those having lower ELs possibly underestimating the necessity of consulting a cardiologist for ACS FU.^38^

### 4.5 Targeted approaches and recent innovations

The link between educational status and healthcare outcomes, alongside utilisation patterns, underscores the broad impact of social determinants on health. This connection signals a need for targeted interventions aimed at enhancing health literacy, improving access to care and bolstering health outcomes across varying educational and socioeconomic groups.^39^ Nurse‐led consultations have been highlighted as a practical approach to achieving this goal. Studies such as the RESPONSE trial^40^ and the NAILED ACS trial^41^ illustrate the effectiveness of nurse‐coordinated programmes in improving lifestyle habits, reducing risk factors and enhancing medication adherence among post‐ACS patients.^42^ Such evidence supports the argument for broader implementation of nurse‐led, medically supported models of care that cater to individual patient needs.

Beyond nurse‐led, medically supported models of care, additional approaches may contribute to more targeted prevention strategies. Recent advances in machine learning (ML) have enabled the integration of multidimensional data, ranging from clinical and imaging information to multi‐omics profiles and socioeconomic indicators such as education or income level, to better identify subgroups of patients at higher risk and guide personalised interventions. ML‐based models have outperformed traditional risk scores in various domains of cardiovascular care, highlighting their potential to support more refined clinical decision‐making.^43^ In parallel, targeting low‐grade systemic inflammation—present in up to 60% of patients with atherosclerotic cardiovascular disease—has emerged as another promising strategy. Notably, elevated CRP levels are disproportionately found among individuals with lower socioeconomic status, reflecting the broader impact of social determinants on chronic inflammation and cardiovascular risk.^44^ Recent findings from Von zur Mühlen et al. emphasise the importance of high‐sensitivity C‐reactive protein (hsCRP) as a robust predictor of adverse cardiovascular events, potentially stronger than LDL cholesterol.^45^ Their review supports routine hsCRP screening and the use of anti‐inflammatory agents such as low‐dose colchicine to address residual inflammatory risk, which remains underdiagnosed and undertreated in current practice. Together, these complementary innovations illustrate how combining algorithmic stratification, biomarker‐driven screening and tailored care delivery may offer new avenues for improving secondary prevention.

### 4.6 Integrated models for vulnerable populations

Building on previous findings identifying low socioeconomic status as a critical factor associated with higher risks of CVD and mortality, and the particular importance of education and occupation over household income,^20^ our study highlights the need for healthcare systems to focus on access to care post‐ACS. Tailored interventions and FU for patients identified with special needs (i.e. low educational level) are essential. Interventions should include accessible, comprehensive cardiac rehabilitation programmes and continued support that adapts to the educational and occupational backgrounds of the patients, thereby mitigating the impact of socioeconomic barriers on health outcomes. This approach not only addresses the immediate disparities in health outcomes based on socioeconomic factors but also enhances long‐term survival and quality of life for these patients.

To ensure that all patients receive appropriate specialist care, establishing a standardized protocol within healthcare facilities that mandates a referral to a cardiologist or appropriate specialist post‐ACS should help ensure consistency. These protocols should include automatic scheduling of FU appointments before hospital discharge, along with clear communication about their importance. Additionally, cardiologists should consider integrating a case management approach where healthcare providers, such as nurses or patient navigators, are tasked with ensuring patients attend their FU visits. These providers can offer support by addressing logistical barriers to care such as transportation, scheduling conflicts and financial concerns, which are particularly significant for patients from lower socioeconomic backgrounds.

In parallel, emerging technologies and innovations offer new possibilities to strengthen the personalization of post‐ACS care. Recent research has demonstrated the utility of machine learning approaches in identifying patients at increased risk of non‐adherence to FU or adverse clinical outcomes, thereby enabling early triage and timely allocation of resources. These tools could support nurses and care teams in prioritising patients for intensified FU and targeted interventions. In addition, targeting low‐grade systemic inflammation, now recognised as a major residual risk factor in secondary prevention, offers another opportunity to stratify risk and guide treatment.

Taken together, these insights support the development of new care pathways that integrate human resources—such as nurses in case management roles—with technological innovations, including predictive algorithms and biomarker‐guided treatment strategies. By leveraging both the relational and technical dimensions of care, such models could contribute to more equitable outcomes and reduce the long‐term burden of cardiovascular disease in socioeconomically disadvantaged populations.

## 5 LIMITATIONS

Several limitations should be acknowledged. First, education was used as a proxy for socioeconomic status, whereas incorporating additional information on income and occupational status would have provided a more comprehensive assessment. However, using EL has shown adequate correlation with income, and by extension an individual's financial ability to access healthcare, as well as intellectual and cultural capacities, which are a causal pathway to detrimental lifestyle habits.^46^ Additionally, EL is not expected to change during the time‐at‐risk for ACS, unlike occupation or income. Second, FU duration might be insufficient to show significant results when the effect could be modest. Third, although this study did not aim at establishing causality, its findings might experience confounding where some unmeasured variables, such as residential area, could potentially impact the observed associations. Lastly, the baseline SPUM‐ACS cohort was a combination of two studies, Biomarkers and ELIPS, with identical inclusion criteria and endpoints; however, only the ELIPS study conducted a 5‐year FU, explaining the difference in sample size at the 5‐year FU. Differences in baseline characteristics are detailed in Table S1 and the association of EL with the incidence of individual components of MACE in Table S2.

## 6 CONCLUSION

This study underscores the importance of EL as a social determinant of cardiovascular health after ACS within the Swiss healthcare system. Despite equitable prescribing of guideline‐recommended therapies, notable disparities were observed across EL in achieving cardiovascular prevention targets, participating in cardiac rehabilitation, and receiving FU care, particularly disadvantaging individuals with lower EL. These findings emphasize the critical role of education as a determinant of health, influencing individuals' ability to engage with preventive care and ultimately clinical outcomes. The persistence of these disparities, even under universal health coverage, underscores the need to address broader social determinants of health in order to mitigate inequities in cardiovascular care and outcomes.

## AUTHOR CONTRIBUTIONS

Maëlle Achard contributed to data analysis and drafted the manuscript. Cédric Follonier, Evelyne Fournier and Mattia Branca conducted the statistical analyses. Dik Heg provided a critical review of the statistical methods. François Mach conceived and designed the study and critically reviewed the manuscript. Baris Gencer co‐wrote the manuscript. David Carballo, David Nanchen, Lorenz Räber, Roland Klingenberg, Stephan Windecker, Thomas F. Lüscher, Christian M. Matter and Nicolas Rodondi critically reviewed the manuscript for important intellectual content.

## CONFLICT OF INTEREST STATEMENT

T.F.L reports receiving research grants to the institution from Abbot, Biosensors, Biotronik, Boston Scientific, Daichi Sankyo, Eli Lilly and Medtronic, and consultant payments from AstraZeneca, Boehringer Ingelheim, Bayer, Merck and Pfizer, MSD, Roche and Servier. C.M.M. reports receiving grants from MSD, AstraZeneca and Roche, and having patents from Mabimmune, CH. S.W. reports receiving research contracts to the institution from Abbott, Biotronik, Boston Scientific, Biosensors, Cordis, Medtronic, St Jude Medical, and speaker fees from Abbott, Biotronik, Boston Scientific, Biosensors, Medtronic, Eli Lilly and AstraZeneca. F.M. has received research grants to the institution from Amgen, AstraZeneca, Eli Lilly, MSD, Novartis, Sanofi and Pfizer, including speaker or consultant fees. Other authors did not report conflicts of interest.

## Supporting information


Figure S1.

Table S1.

## Data Availability

The data that support the findings of this study are available from the corresponding author upon reasonable request.

## References

[1] Schultz WM , Kelli HM , Lisko JC , et al. Socioeconomic status and cardiovascular outcomes: challenges and interventions. Circulation. 2018;137(20):2166‐2178. 29760227 10.1161/CIRCULATIONAHA.117.029652 PMC5958918

[2] Stringhini S , Carmeli C , Jokela M , et al. Socioeconomic status and the 25 × 25 risk factors as determinants of premature mortality: a multicohort study and meta‐analysis of 1·7 million men and women. Lancet. 2017;389(10075):1229‐1237. 28159391 10.1016/S0140-6736(16)32380-7 PMC5368415

[3] Laine JE , Baltar VT , Stringhini S , et al. Reducing socio‐economic inequalities in all‐cause mortality: a counterfactual mediation approach. Int J Epidemiol. 2020;49(2):497‐510. 31855265 10.1093/ije/dyz248 PMC7266549

[4] Jaquet E , Gencer B , Auer R , et al. Association between income and control of cardiovascular risk factors after acute coronary syndromes: an observational study. Swiss Med Wkly. 2019;149(1516):w20049. 30994923 10.4414/smw.2019.20049

[5] McDonagh TA , Metra M , Adamo M , et al. Corrigendum to: 2021 ESC guidelines for the diagnosis and treatment of acute and chronic heart failure: developed by the task force for the diagnosis and treatment of acute and chronic heart failure of the European Society of Cardiology (ESC) with the special contribution of the heart failure association (HFA) of the ESC. Eur Heart J. 2021;42(48):4901. 34649282 10.1093/eurheartj/ehab670

[6] Kaplan GA , Keil JE . Socioeconomic factors and cardiovascular disease: a review of the literature. Circulation. 1993;88(4):1973‐1998. 8403348 10.1161/01.cir.88.4.1973

[7] Galobardes B , Shaw M , Lawlor DA , Lynch JW , Davey Smith G . Indicators of socioeconomic position (part 1). J Epidemiol Community Health. 2006;60(1):7. 10.1136/jech.2004.023531 PMC2465546 16361448

[8] Rosengren A , Smyth A , Rangarajan S , et al. Socioeconomic status and risk of cardiovascular disease in 20 low‐income, middle‐income, and high‐income countries: the prospective urban rural epidemiologic (PURE) study. Lancet Glob Health. 2019;7(6):e748‐e760. 31028013 10.1016/S2214-109X(19)30045-2

[9] Bruthans J , Mayer O Jr , De Bacquer D , et al. Educational level and risk profile and risk control in patients with coronary heart disease. Eur J Prev Cardiol. 2016;23(8):881‐890. 26283652 10.1177/2047487315601078

[10] OFSP O Fédéral de la Santé Publique . Stratégie nationale Prévention des maladies non transmissibles. [cited 2021 Feb 20]. Available from: https://www.bag.admin.ch/bag/fr/home/strategie‐und‐politik/nationale‐gesundheitsstrategien/strategie‐nicht‐uebertragbare‐krankheiten.html

[11] RS 832.112.31 – Ordonnance du DFI du 29 septembr… | Fedlex. [cited 2024 Apr 3]. Available from: https://www.fedlex.admin.ch/eli/cc/1995/4964_4964_4964/fr

[12] Timmis A , Vardas P , Townsend N , et al. European Society of Cardiology: cardiovascular disease statistics 2021. Eur Heart J. 2022;43:716‐799. 35016208 10.1093/eurheartj/ehab892

[13] de Pietro C , Camenzind P , Sturny I , et al. The performance of the Swiss health system: good results but high costs: Wilm Quentin. Eur J Pub Health. 2015;25(suppl_3):ckv174‐080.

[14] Smith SC , Benjamin EJ , Bonow RO , et al. AHA/ACCF secondary prevention and risk reduction therapy for patients with coronary and other atherosclerotic vascular disease: 2011 update: a guideline from the American Heart Association and American College of Cardiology Foundation endorsed by the world heart federation and the preventive cardiovascular nurses association. J Am Coll Cardiol. 2011;58(23):2432‐2446. 22055990 10.1016/j.jacc.2011.10.824

[15] Statistique O fédéral de la. Office fédéral de la statistique . Le système d'enseignement en Suisse (simplifié) | Image. 2015 [cited 2024 Apr 10]. Available from: https://www.bfs.admin.ch/asset/fr/223674

[16] Piepoli MF , Hoes AW , Agewall S , et al. 2016 European guidelines on cardiovascular disease prevention in clinical practice: the sixth joint task force of the European Society of Cardiology and Other Societies on cardiovascular disease prevention in clinical practice (constituted by representatives of 10 societies and by invited experts)developed with the special contribution of the European Association for Cardiovascular Prevention & rehabilitation (EACPR). Eur Heart J. 2016;37(29):2315‐2381. 27222591 10.1093/eurheartj/ehw106 PMC4986030

[17] Perk J , De Backer G , Gohlke H , et al. European Guidelines on cardiovascular disease prevention in clinical practice (version 2012). The Fifth Joint Task Force of the European Society of Cardiology and Other Societies on Cardiovascular Disease Prevention in Clinical Practice (constituted by representatives of nine societies and by invited experts). Eur Heart J. 2012;33:1635‐1701. doi:10.1093/eurheartj/ehs092 22555213

[18] Sampson M , Ling C , Sun Q , et al. A new equation for calculation of low‐density lipoprotein cholesterol in patients with Normolipidemia and/or hypertriglyceridemia. JAMA Cardiol. 2020;5(5):540‐548. 32101259 10.1001/jamacardio.2020.0013 PMC7240357

[19] Khaing W , Vallibhakara SA , Attia J , McEvoy M , Thakkinstian A . Effects of education and income on cardiovascular outcomes: a systematic review and meta‐analysis. Eur J Prev Cardiol. 2017;24(10):1032‐1042. 28406328 10.1177/2047487317705916

[20] Hahad O , Gilan DA , Chalabi J , et al. Cumulative social disadvantage and cardiovascular disease burden and mortality. Eur J Prev Cardiol. 2024;31(1):40‐48. 37721449 10.1093/eurjpc/zwad264

[21] Deraz O , Kab S , Touvier M , et al. Life's essential 8 cardiovascular health status of 18–69‐year‐old individuals in France. Am J Prev Cardiol. 2025;22:100981. 40242362 10.1016/j.ajpc.2025.100981 PMC12003004

[22] Pérez‐Miguel E , Trias‐Llimós S . Educational inequalities in cardiovascular mortality in Spanish regions (2016–2021). Gac Sanit. 2025;39:102458. 39978007 10.1016/j.gaceta.2025.102458

[23] Bonaccio M , Di Castelnuovo A , Pounis G , et al. High adherence to the Mediterranean diet is associated with cardiovascular protection in higher but not in lower socioeconomic groups: prospective findings from the Moli‐sani study. Int J Epidemiol. 2017;46(5):1478‐1487. 29040542 10.1093/ije/dyx145

[24] Wang N , Jia X , Fan Z , et al. Educational inequalities in cardiovascular diseases and their mediating factors across different generations: a prospective cohort study. Eur Heart J Qual Care Clin Outcomes. 2025;qcaf010. 10.1093/ehjqcco/qcaf010 40036661

[25] Ohm J , Skoglund PH , Häbel H , et al. Association of Socioeconomic Status with Risk Factor Target Achievements and use of secondary prevention after myocardial infarction. JAMA Netw Open. 2021;4(3):e211129. 33688966 10.1001/jamanetworkopen.2021.1129 PMC7948055

[26] Bachmann JM , Huang S , Gupta DK , et al. Association of Neighborhood Socioeconomic Context with Participation in cardiac rehabilitation. J Am Heart Assoc. 2017;6(10):e006260. 29021267 10.1161/JAHA.117.006260 PMC5721841

[27] Yusuf S , Joseph P , Rangarajan S , et al. Modifiable risk factors, cardiovascular disease, and mortality in 155 722 individuals from 21 high‐income, middle‐income, and low‐income countries (PURE): a prospective cohort study. Lancet. 2020;395(10226):795‐808. 31492503 10.1016/S0140-6736(19)32008-2 PMC8006904

[28] Panuccio G , Carabetta N , Torella D , De Rosa S . Percutaneous coronary revascularization versus medical therapy in chronic coronary syndromes: an updated meta‐analysis of randomized controlled trials. Eur J Clin Investig. 2024;54(12):e14303. 39166630 10.1111/eci.14303

[29] Schröder SL , Richter M , Schröder J , Frantz S , Fink A . Socioeconomic inequalities in access to treatment for coronary heart disease: a systematic review. Int J Cardiol. 2016;219:70‐78. 27288969 10.1016/j.ijcard.2016.05.066

[30] Gil‐Guillen VF , Balsa A , Bernárdez B , et al. Medication non‐adherence in rheumatology, oncology and cardiology: a review of the literature of risk factors and potential interventions. Int J Environ Res Public Health. 2022;19(19):12036. 36231341 10.3390/ijerph191912036 PMC9564665

[31] Mackenbach JP . Re‐thinking health inequalities. Eur J Pub Health. 2020;30(4):615. 32558893 10.1093/eurpub/ckaa001 PMC7445033

[32] Maqsood MH , Nguyen R , Chang R , et al. Unfavorable social determinants of health and mortality risk by cardiovascular disease status: findings from a National Study of United States adults. Am Heart J. 2024;267:95‐100. 38071003 10.1016/j.ahj.2023.10.006

[33] Teshale AB , Htun HL , Owen A , et al. The role of social determinants of health in cardiovascular diseases: an umbrella review. J Am Heart Assoc. 2023;12(13):e029765. 37345825 10.1161/JAHA.123.029765 PMC10356094

[34] McMaughan DJ , Oloruntoba O , Smith ML . Socioeconomic status and access to healthcare: interrelated drivers for healthy aging. Front Public Health. 2020;8:231. 32626678 10.3389/fpubh.2020.00231 PMC7314918

[35] Lueckmann SL , Hoebel J , Roick J , et al. Socioeconomic inequalities in primary‐care and specialist physician visits: a systematic review. Int J Equity Health. 2021;20(1):58. 33568126 10.1186/s12939-020-01375-1 PMC7874661

[36] Berkman ND , Sheridan SL , Donahue KE , Halpern DJ , Crotty K . Low health literacy and health outcomes: an updated systematic review. Ann Intern Med. 2011;155(2):97‐107. 21768583 10.7326/0003-4819-155-2-201107190-00005

[37] Gulliford M , Figueroa‐Munoz J , Morgan M , et al. What does ‘access to health care’ mean? J Health Serv Res Policy. 2002;7:186‐188. doi:10.1258/135581902760082517 12171751

[38] Wardle J , Steptoe A . Socioeconomic differences in attitudes and beliefs about healthy lifestyles. J Epidemiol Community Health. 2003;57(6):440‐443. 12775791 10.1136/jech.57.6.440 PMC1732468

[39] Visseren FLJ , Mach F , Smulders YM , et al. 2021 ESC guidelines on cardiovascular disease prevention in clinical practice: developed by the task force for cardiovascular disease prevention in clinical practice with representatives of the European Society of Cardiology and 12 medical societies with the special contribution of the European Association of Preventive Cardiology (EAPC). Eur Heart J. 2021;42(34):3227‐3337.34458905

[40] Jorstad HT , von Birgelen C , Alings AMW , et al. Effect of a nurse‐coordinated prevention programme on cardiovascular risk after an acute coronary syndrome: main results of the RESPONSE randomised trial. Heart. 2013;99(19):1421‐1430. 23813851 10.1136/heartjnl-2013-303989 PMC3786610

[41] Henriksson R , Huber D , Mooe T . Nurse‐led, telephone‐based follow‐up after acute coronary syndrome yields improved risk factors after 36 months: the randomized controlled NAILED‐ACS trial. Sci Rep. 2021;11:17693. 34489516 10.1038/s41598-021-97239-x PMC8421439

[42] Corones‐Watkins K , Cooke M , Theobald K , et al. Effectiveness of nurse‐led clinics in the early discharge period after percutaneous coronary intervention: a systematic review. Aust Crit Care. 2021;34(5):510‐517. 33272768 10.1016/j.aucc.2020.10.012

[43] Wang Y , Aivalioti E , Stamatelopoulos K , et al. Machine learning in cardiovascular risk assessment: towards a precision medicine approach. Eur J Clin Investig. 2025;55(S1):e70017. 40191920 10.1111/eci.70017

[44] Liu RS , Aiello AE , Mensah FK , et al. Socioeconomic status in childhood and C‐reactive protein in adulthood: a systematic review and meta‐analysis. J Epidemiol Community Health. 2017;71(8):817‐826. 28490476 10.1136/jech-2016-208646 PMC5843476

[45] Arnold N , Koenig W . Inflammation in atherosclerotic cardiovascular disease: from diagnosis to treatment. Eur J Clin Investig. 2025;55:e70020. 40055964 10.1111/eci.70020 PMC12169090

[46] Marmot M , Ryff CD , Bumpass LL , Shipley M , Marks NF . Social inequalities in health: next questions and converging evidence. Soc Sci Med. 1997;44(6):901‐910. 9080570 10.1016/s0277-9536(96)00194-3

