# Supplementary material for: Educational level, clinical outcomes and quality of care in a Swiss cohort of patients with acute coronary syndromes
Source: Eur J Clin Invest. 2025 Jul 26;55(12):e70097. doi: 10.1111/eci.70097 (PMC12621298; doi:10.1111/eci.70097)
Supplement: Supplementary file 1 — Figure S1. [file ECI-55-e70097-s001.docx]

**Supplementary Figure 1.** Study flowchart

**
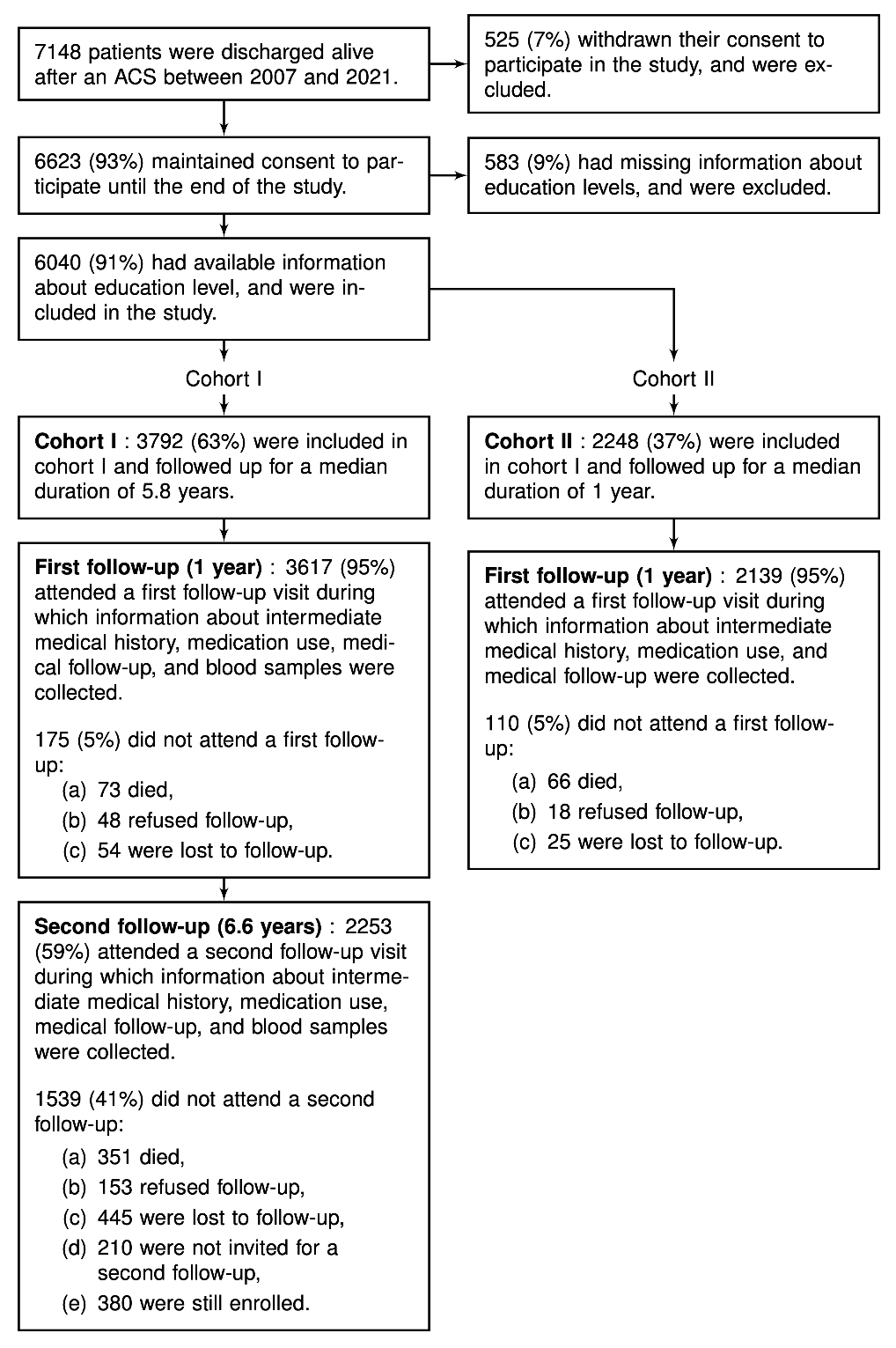
**

**Supplementary Figure 2**. Directed acyclic graph (DAG) for the relationship between education levels and cardiovascular therapies prescriptions, achievement of secondary prevention targets and clinical outcomes after ACS.


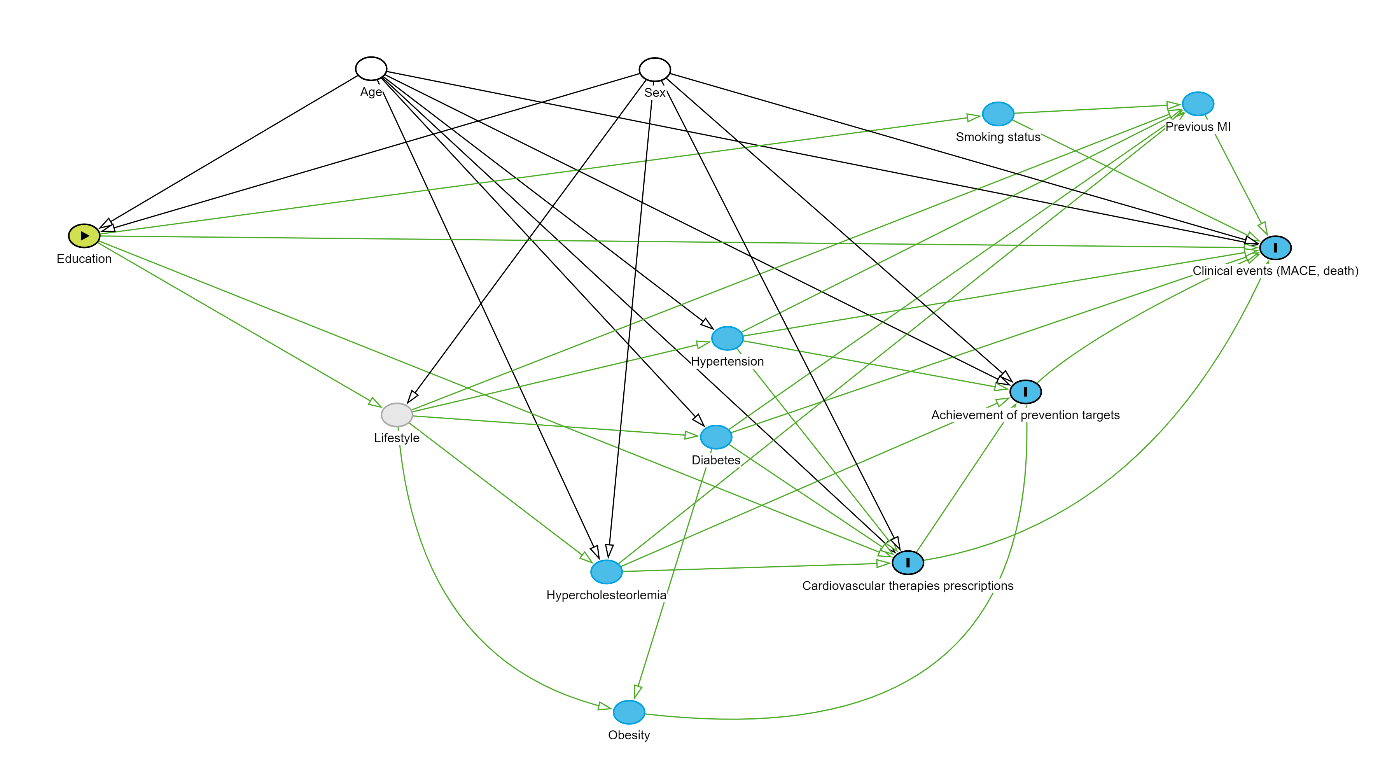


*Legends: green node with a triangle = exposure, blue node with a line = outcome, white node = adjustment variables, blue node = ancestor of the outcome, red node = ancestor of exposure and outcome, green path = causal path, purple path = biasing path, black path = other path.*
