# Supplementary material for: Educational level, clinical outcomes and quality of care in a Swiss cohort of patients with acute coronary syndromes
Source: Eur J Clin Invest. 2025 Jul 26;55(12):e70097. doi: 10.1111/eci.70097 (PMC12621298; doi:10.1111/eci.70097)
Supplement: Supplementary file 2 — Table S1. [file ECI-55-e70097-s002.docx]

**Supplementary Table 1**. Baseline characteristics according to study participation

| **Characteristic** | **Overall**  **(N = 6040)** | **Cohort II**  **(Biomarke substudy, N = 2248)** | **Cohort I**  **(ELIPS substudy, N = 3792)** | **P value** |
| --- | --- | --- | --- | --- |
| **Sociodemographics** |  |  |  |  |
| Age | 63 (12) | 64 (12) | 62 (12) | <0.001 |
| Male sex | 4869/6040 (81%) | 1819/2248 (81%) | 3050/3792 (80%) | 0.646 |
| Education |  |  |  | <0.001 |
| EL4 | 1060/6040 (18%) | 286/2248 (13%) | 774/3792 (20%) |  |
| EL3 | 847/6040 (14%) | 247/2248 (11%) | 600/3792 (16%) |  |
| EL2 | 3118/6040 (52%) | 1425/2248 (63%) | 1693/3792 (45%) |  |
| EL1 | 1015/6040 (17%) | 290/2248 (13%) | 725/3792 (19%) |  |
| BMI (kg/m^2^) | 27.0 (4.3) | 27.1 (4.4) | 27.0 (4.3) | 0.652 |
| Caucasian ethnicity | 5737/6001 (96%) | 2170/2238 (97%) | 3567/3763 (95%) | <0.001 |
| Married or in partnership | 4024/6034 (67%) | 1622/2244 (72%) | 2402/3790 (63%) | <0.001 |
| Living alone | 1469/6025 (24%) | 521/2240 (23%) | 948/3785 (25%) | 0.118 |
| Working status |  |  |  | <0.001 |
| *Full time* | 2549/6002 (42%) | 924/2222 (42%) | 1625/3780 (43%) |  |
| *Part time* | 506/6002 (8.4%) | 152/2222 (6.8%) | 354/3780 (9.4%) |  |
| *No employment/retired* | 2947/6002 (49%) | 1146/2222 (52%) | 1801/3780 (48%) |  |
| **Medical history** |  |  |  |  |
| Obesity (BMI ≥30kg/m^2^) | 1283/5973 (21%) | 510/2206 (23%) | 773/3767 (21%) | 0.018 |
| Current smoking | 2417/6025 (40%) | 818/2234 (37%) | 1599/3791 (42%) | <0.001 |
| Daily alcohol consumption | 1536/5899 (26%) | 455/2173 (21%) | 1081/3726 (29%) | <0.001 |
| Diabetes | 1009/6039 (17%) | 400/2248 (18%) | 609/3791 (16%) | 0.082 |
| Hypertension | 3231/6037 (54%) | 1283/2247 (57%) | 1948/3790 (51%) | <0.001 |
| Stroke or transient ischemic attack | 228/6038 (3.8%) | 87/2248 (3.9%) | 141/3790 (3.7%) | 0.768 |
| Peripheral vascular disease | 309/6038 (5.1%) | 122/2248 (5.4%) | 187/3790 (4.9%) | 0.401 |
| Heart failure | 77/6038 (1.3%) | 30/2248 (1.3%) | 47/3790 (1.2%) | 0.752 |
| End-stage kidney disease | 37/6038 (0.6%) | 11/2248 (0.5%) | 26/3790 (0.7%) | 0.344 |
| Lung disease (COPD or asthma) | 209/6031 (3.5%) | 93/2247 (4.1%) | 116/3784 (3.1%) | 0.028 |
| Liver disease | 38/6038 (0.6%) | 11/2248 (0.5%) | 27/3790 (0.7%) | 0.289 |
| Malignancy | 460/6034 (7.6%) | 188/2248 (8.4%) | 272/3786 (7.2%) | 0.095 |
| Systemic inflammatory disease | 155/6038 (2.6%) | 62/2248 (2.8%) | 93/3790 (2.5%) | 0.470 |
| **Lipid profile at baseline** |  |  |  |  |
| Total cholesterol | 5.0 (4.2, 5.7) | 4.8 (4.0, 5.6) | 5.1 (4.3, 5.9) | <0.001 |
| LDL-C | 3.6 (2.8, 4.4) | 3.4 (2.7, 4.2) | 3.7 (2.9, 4.5) | <0.001 |
| HDL-C | 1.1 (0.9, 1.4) | 1.1 (1.0, 1.4) | 1.1 (0.9, 1.4) | 0.140 |
| Triglycerides | 1.1 (0.7, 1.7) | 0.9 (0.6, 1.4) | 1.2 (0.8, 1.9) | <0.001 |
| **Medication at baseline** |  |  |  |  |
| Aspirin | 1566/6000 (26%) | 604/2224 (27%) | 962/3776 (25%) | 0.152 |
| Anti-P2Y12 | 370/4694 (7.9%) | 139/1986 (7.0%) | 231/2708 (8.5%) | 0.054 |
| Statins | 1549/5990 (26%) | 573/2219 (26%) | 976/3771 (26%) | 0.960 |
| Beta-blockers | 1219/5982 (20%) | 519/2212 (23%) | 700/3770 (19%) | <0.001 |
| Renin-angiotensin system inhibitors | 1966/5975 (33%) | 777/2213 (35%) | 1189/3762 (32%) | 0.005 |
| Oral antidiabetics (if diabetics) | 667/995 (67%) | 253/389 (65%) | 414/606 (68%) | 0.283 |
| Insulin (if diabetics) | 268/996 (27%) | 113/389 (29%) | 155/607 (26%) | 0.223 |
| **Clinical data** |  |  |  |  |
| Type of ACS |  |  |  | <0.001 |
| *NSTEMI* | 2508/6040 (42%) | 972/2248 (43%) | 1536/3792 (41%) |  |
| *STEMI* | 3278/6040 (54%) | 1208/2248 (54%) | 2070/3792 (55%) |  |
| *Unstable angina* | 247/6040 (4.1%) | 68/2248 (3.0%) | 179/3792 (4.7%) |  |
| *Undertermined* | 7/6040 (0.1%) | 0/2248 (0%) | 7/3792 (0.2%) |  |
| Kilipp class II-IV | 642/5793 (11%) | 300/2157 (14%) | 342/3636 (9.4%) | <0.001 |
| Discharge destination |  |  |  | <0.001 |
| *Home* | 3390/6024 (56%) | 826/2237 (37%) | 2564/3787 (68%) |  |
| *Other hospital* | 2008/6024 (33%) | 1129/2237 (50%) | 879/3787 (23%) |  |
| *Rehabilitation center* | 626/6024 (10%) | 282/2237 (13%) | 344/3787 (9.1%) |  |
| Categorical variables are expressed as percentages (count with percentage), while continuous variables are reported as either mean ± standard deviation or median with interquartile range. BMI was missing for 67 participants, total cholesterol for 302, LDL-C for 405, HDL-C for 392, and triglycerides for 376. P values were determined using Chi-squared tests for categorical variables, Student’s T-test for normally distributed continuous variables, and Kruskal-Wallis test for non-normally distributed continuous variables. *Abbreviations: EL = education level, BMI = body mass index, COPD = chronic obstructive pulmonary disease, LDL-C = low-density lipoprotein cholesterol, HDL-C = high-density lipoprotein cholesterol* | | | | |

**Supplementary Table 2**. Association of education levels with the incidence of individual components of MACE

|  |  | **Model 1 (unadjusted)** | | **Model 2**  **(adjusted for age and sex)** | | **Model 3**  **(adjusted for age, sex and traditional cardiovascular risk factors)** | |
| --- | --- | --- | --- | --- | --- | --- | --- |
|  | n (%) | Unadj. HR (95%CI) | p-value | Adj. HR (95%CI) | p-value | Adj. HR (95%CI) | p-value |
| Myocardial infarction |  |  |  |  |  |  |  |
| EL4 | 22 (2%) | ref. |  | ref. |  | ref. |  |
| EL3 | 23 (3%) | 1.33 (0.74, 2.38) | 0.343 | 1.38 (0.77, 2.49) | 0.276 | 1.40 (0.77, 2.55) | 0.275 |
| EL2 | 80 (3%) | 1.24 (0.77, 1.98) | 0.376 | 1.23 (0.77, 1.98) | 0.388 | 1.17 (0.72, 1.91) | 0.524 |
| EL1 | 19 (2%) | 0.91 (0.49, 1.68) | 0.761 | 0.91 (0.49, 1.69) | 0.755 | 0.76 (0.40, 1.45) | 0.398 |
| Stroke or TIA |  |  |  |  |  |  |  |
| EL4 | 9 (1%) | ref. |  | ref. |  | ref. |  |
| EL3 | 12 (1%) | 1.69 (0.71, 4.00) | 0.236 | 1.78 (0.75, 4.24) | 0.192 | 1.76 (0.74, 4.20) | 0.202 |
| EL2 | 26 (1%) | 0.99 (0.46, 2.10) | 0.970 | 0.97 (0.45, 2.07) | 0.930 | 0.83 (0.38, 1.80) | 0.636 |
| EL1 | 19 (2%) | 2.24 (1.01, 4.95) | 0.046 | 2.16 (0.96, 4.86) | 0.063 | 1.96 (0.86, 4.46) | 0.108 |
| Coronary revascularization |  |  |  |  |  |  |  |
| EL4 | 37 (3%) | ref. |  | ref. |  | ref. |  |
| EL3 | 57 (7%) | 1.99 (1.31, 3.01) | 0.001 | 2.02 (1.34, 3.06) | <0.001 | 2.00 (1.32, 3.03) | 0.001 |
| EL2 | 171 (5%) | 1.60 (1.12, 2.28) | 0.010 | 1.60 (1.12, 2.28) | 0.010 | 1.51 (1.06, 2.17) | 0.024 |
| EL1 | 46 (5%) | 1.32 (0.86, 2.04) | 0.205 | 1.33 (0.86, 2.06) | 0.206 | 1.18 (0.75, 1.84) | 0.474 |
| Cardiovascular death |  |  |  |  |  |  |  |
| EL4 | 8 (1%) | ref. |  | ref. |  | ref. |  |
| EL3 | 17 (2%) | 2.68 (1.16, 6.21) | 0.021 | 2.98 (1.28, 6.91) | 0.011 | 3.08 (1.32, 7.17) | 0.009 |
| EL2 | 49 (2%) | 2.09 (0.99, 4.42) | 0.053 | 2.02 (0.95, 4.27) | 0.066 | 1.67 (0.78, 3.56) | 0.187 |
| EL1 | 20 (2%) | 2.63 (1.16, 5.96) | 0.021 | 2.54 (1.10, 5.84) | 0.028 | 1.96 (0.84, 4.57) | 0.121 |
| Data are presented as count and percentage. Hazard ratios with 95% confidence interval are presented unadjusted (model 1), adjusted for age and sex (model 2), and for age, sex, BMI, history of hypertension, smoking status, diabetes, hypercholesterolemia, and previous MI (model 3). | | | | | | | |

**Supplementary Table 3**. Unadjusted (model 1) and adjusted (model 3) associations of attendance of cardiac rehabilitation and medical follow-up during the first-year post ACS with education levels

|  | **Model 1**  **(unadjusted)** | | | | | | **Model 3**  **(adjusted for age, sex and traditional risk factors)** | | | | | |
| --- | --- | --- | --- | --- | --- | --- | --- | --- | --- | --- | --- | --- |
|  | EL 1 vs. EL4 | | EL2 vs. EL4 | | EL3 vs. EL4 | | EL 1 vs. EL4 | | EL2 vs. EL4 | | EL3 vs. EL4 | |
|  | Unadj OR (95%CI)  [RMD (95% CI)] | p-value | Unadj. OR (95%CI)  [RMD (95% CI)] | p-value | Unadj. OR (95%CI)  [RMD (95% CI)] | p-value | Adj. OR (95%CI)  [AME (95% CI)] | p-value | Adj. OR (95%CI)  [AME (95% CI)] | p-value | Adj. OR (95%CI)  [AME (95% CI)] | p-value |
| Attendance to cardiac rehabilitation | 0.58 (0.48, 0.71) [-10.6 (-14.6, -6.7)] | <0.001 | 0.82 (0.69, 0.97) [-3.7 (-6.7, -0.7)] | 0.020 | 0.94 (0.75, 1.17) [-1.1 (-5.0, 2.8)] | 0.573 | 0.69 (0.57, 0.85) [-7.1 (-11.0, -3.2)] | <0.001 | 0.87 (0.74, 1.03) [-2.5 (-5.5, 0.5)] | 0.112 | 0.91 (0.73, 1.13) [-1.7 (-5.7, 2.2)] | 0.384 |
| Any medical follow-up | 0.69 (0.28, 1.65) [-0.4 (-1.4, 0.6)] | 0.410 | 0.63 (0.29, 1.24) [-0.5 (-1.3, 0.2)] | 0.210 | 0.37 (0.16, 0.79) [-1.6 (-2.8, -0.3)] | 0.014 | 0.67 (0.26, 1.69) [-0.4 (-1.4, 0.5)] | 0.391 | 0.55 (0.26, 1.19) [-0.7 (-1.4, 0.1)] | 0.128 | 0.34 (0.15, 0.77) [-1.6 (-2.8, -0.4)] | 0.010 |
| Medical follow-up with a general practitioner | 2.34 (1.68, 3.31) [6.9 (4.3, 9.6)] | <0.001 | 2.00 (1.57, 2.54) [6.0 (3.7, 8.3)] | <0.001 | 1.63 (1.19, 2.27) [4.6 (1.7, 7.5)] | 0.003 | 1.99 (1.41, 2.81) [5.6 (2.9, 8.3)] | <0.001 | 1.85 (1.45, 2.36) [5.2 (2.9, 7.5)] | <0.001 | 1.61 (1.16, 2.22) [4.2 (1.4, 7.0)] | 0.004 |
| Medical follow-up with a cardiologist | 0.59 (0.46, 0.77) [-6.7 (-9.9, -3.4)] | <0.001 | 0.54 (0.43, 0.67) [-8.1 (-10.6, -5.5)] | <0.001 | 1.11 (0.82, 1.51) [1.0 (-2.0, 4.1)] | 0.505 | 0.67 (0.51, 0.87) [-5.0 (-8.3, -1.7)] | 0.003 | 0.57 (0.46, 0.71) [-7.4 (-9.9, -4.8)] | <0.001 | 1.08 (0.79, 1.46) [0.8 (-2.4, 3.9)] | 0.637 |
| Unadjusted odds ratios, adjusted odds ratios, raw mean difference, and average marginal effect with 95% confidence interval are presented. Model 1 is unadjusted, and model 3 is adjusted for age, sex and traditional cardiovascular risk factors (hypertension, body mass index, smoking status, diabetes, previous myocardial infarction and hypercholesterolemia). Results from model 2 (adjusted for age and sex) are presented in the manuscript. *Abbreviations: OR = odds ratio, RMD = raw mean difference, CI = confidence interval* | | | | | | | | | | | | |

**Supplementary Table 4**. Unadjusted (model 1) and adjusted (model 3) associations of prescription of recommend cardiovascular therapies with education level

|  | **Model 1**  **(unadjusted)** | | | | | | **Model 3**  **(adjusted for age, sex and traditional risk factors)** | | | | | |
| --- | --- | --- | --- | --- | --- | --- | --- | --- | --- | --- | --- | --- |
|  | EL 1 vs. EL4 | | EL2 vs. EL4 | | EL3 vs. EL4 | | EL 1 vs. EL4 | | EL2 vs. EL4 | | EL3 vs. EL4 | |
|  | Unadj OR (95%CI)  [RMD (95% CI)] | p-value | Unadj. OR (95%CI)  [RMD (95% CI)] | p-value | Unadj. OR (95%CI)  [RMD (95% CI)] | p-value | Adj. OR (95%CI)  [AME (95% CI)] | p-value | Adj. OR (95%CI)  [AME (95% CI)] | p-value | Adj. OR (95%CI)  [AME (95% CI)] | p-value |
| **Discharge** |  |  |  |  |  |  |  |  |  |  |  |  |
| Aspirin | 2.41 (0.80, 8.80) [0.5 (-0.1, 1.2)] | 0.139 | 1.28 (0.58, 2.63) [0.2 (-0.4, 0.9)] | 0.515 | 1.14 (0.44, 3.16) [0.1 (-0.7, 1.0)] | 0.787 | 2.84 (0.84, 9.63) [0.6 (-0.1, 1.3)] | 0.094 | 1.19 (0.54, 2.61) [0.1 (-0.5, 0.8)] | 0.659 | 1.09 (0.39, 3.08) [0.1 (-0.8, 1.0)] | 0.867 |
| Anti-P2Y12 | 1.29 (0.65, 2.63) [0.4 (-0.7, 1.5)] | 0.476 | 0.94 (0.54, 1.55) [-0.1 (-1.1, 0.8)] | 0.813 | 1.17 (0.58, 2.44) [0.3 (-0.9, 1.4)] | 0.661 | 1.65 (0.80, 3.38) [0.8 (-0.4, 1.9)] | 0.175 | 1.07 (0.63, 1.82) [0.1 (-0.9, 1.2)] | 0.813 | 1.28 (0.62, 2.65) [0.4 (-0.8, 1.7)] | 0.509 |
| Statins | 0.77 (0.43, 1.37) [-0.6 (-1.9, 0.7)] | 0.380 | 1.14 (0.68, 1.84) [0.2 (-0.7, 1.2)] | 0.611 | 0.92 (0.50, 1.73) [-0.2 (-1.5, 1.1)] | 0.802 | 0.89 (0.49, 1.60) [-0.3 (-1.6, 1.1)] | 0.695 | 1.25 (0.76, 2.07) [0.4 (-0.6, 1.5)] | 0.384 | 1.00 (0.53, 1.89) [-0.0 (-1.4, 1.4)] | 0.994 |
| ACEi/ARB | 1.15 (0.87, 1.51) [1.3 (-1.4, 4.0)] | 0.330 | 1.01 (0.81, 1.25) [0.1 (-2.2, 2.3)] | 0.941 | 1.19 (0.89, 1.59) [1.7 (-1.1, 4.5)] | 0.249 | 1.10 (0.83, 1.47) [1.0 (-1.8, 3.7)] | 0.495 | 0.97 (0.78, 1.21) [-0.3 (-2.5, 1.9)] | 0.782 | 1.15 (0.86, 1.55) [1.4 (-1.4, 4.1)] | 0.341 |
| Beta-blockers | 1.11 (0.89, 1.39) [1.6 (-1.8, 4.9)] | 0.353 | 0.88 (0.74, 1.04) [-2.1 (-4.9, 0.7)] | 0.147 | 1.04 (0.82, 1.31) [0.6 (-3.0, 4.1)] | 0.754 | 1.02 (0.81, 1.28) [0.3 (-3.2, 3.7)] | 0.868 | 0.85 (0.71, 1.01) [-2.7 (-5.5, 0.1)] | 0.065 | 1.00 (0.80, 1.26) [0.0 (-3.5, 3.6)] | 0.983 |
| Oral antidiabetics (if diabetic) | 0.63 (0.39, 1.01) [-9.8 (-19.6, -0.0)] | 0.057 | 0.80 (0.51, 1.21) [-4.7 (-13.2, 3.8)] | 0.298 | 0.70 (0.40, 1.23) [-7.5 (-19.3, 4.3)] | 0.212 | 0.64 (0.39, 1.06) [-9.3 (-19.5, 0.9)] | 0.083 | 0.78 (0.50, 1.21) [-5.1 (-13.8, 3.6)] | 0.266 | 0.67 (0.38, 1.18) [-8.4 (-20.3, 3.5)] | 0.168 |
| Insulin (if diabetic) | 0.80 (0.51, 1.25) [-5.2 (-15.6, 5.2)] | 0.327 | 0.93 (0.63, 1.39) [-1.7 (-11.0, 7.7)] | 0.726 | 0.82 (0.48, 1.40) [-4.6 (-17.0, 7.8)] | 0.468 | 0.80 (0.50, 1.27) [-5.2 (-16.1, 5.7)] | 0.346 | 0.94 (0.63, 1.41) [-1.4 (-11.0, 8.2)] | 0.773 | 0.84 (0.49, 1.45) [-3.9 (-16.5, 8.6)] | 0.541 |
| **First follow-up (1 year)** |  |  |  |  |  |  |  |  |  |  |  |  |
| Aspirin | 0.89 (0.55, 1.43) [-0.4 (-2.1, 1.2)] | 0.618 | 0.87 (0.58, 1.27) [-0.5 (-1.8, 0.8)] | 0.473 | 0.78 (0.48, 1.27) [-0.9 (-2.7, 0.9)] | 0.315 | 0.95 (0.57, 1.55) [-0.2 (-1.8, 1.4)] | 0.824 | 0.88 (0.59, 1.31) [-0.5 (-1.8, 0.9)] | 0.515 | 0.67 (0.41, 1.09) [-1.6 (-3.5, 0.4)] | 0.106 |
| Anti-P2Y12 | 1.14 (0.91, 1.44) [2.1 (-1.5, 5.8)] | 0.259 | 1.33 (1.10, 1.60) [4.3 (1.3, 7.2)] | 0.003 | 0.98 (0.77, 1.24) [-0.4 (-4.3, 3.5)] | 0.838 | 1.14 (0.89, 1.44) [2.0 (-1.8, 5.7)] | 0.299 | 1.29 (1.07, 1.56) [3.9 (0.9, 6.8)] | 0.008 | 0.96 (0.75, 1.21) [-0.8 (-4.7, 3.2)] | 0.707 |
| Statins | 0.94 (0.68, 1.30) [-0.5 (-2.9, 1.9)] | 0.701 | 1.17 (0.89, 1.53) [1.0 (-0.8, 2.9)] | 0.258 | 0.88 (0.63, 1.24) [-0.9 (-3.5, 1.6)] | 0.467 | 1.01 (0.72, 1.42) [0.1 (-2.4, 2.5)] | 0.967 | 1.17 (0.89, 1.55) [1.1 (-0.9, 3.0)] | 0.261 | 0.87 (0.62, 1.23) [-1.0 (-3.6, 1.6)] | 0.440 |
| ACEi/ARB | 1.34 (1.08, 1.67) [4.8 (1.2, 8.4)] | 0.009 | 1.24 (1.04, 1.47) [3.6 (0.7, 6.6)] | 0.014 | 1.15 (0.92, 1.44) [2.4 (-1.4, 6.3)] | 0.217 | 1.18 (0.94, 1.47) [2.6 (-1.0, 6.3)] | 0.152 | 1.12 (0.94, 1.33) [1.9 (-1.0, 4.8)] | 0.192 | 1.11 (0.89, 1.38) [1.7 (-2.0, 5.4)] | 0.366 |
| Beta-blockers | 1.40 (1.15, 1.72) [6.6 (2.7, 10.4)] | 0.001 | 1.33 (1.14, 1.56) [5.6 (2.4, 8.9)] | <0.001 | 1.09 (0.89, 1.34) [1.8 (-2.4, 6.0)] | 0.412 | 1.24 (1.00, 1.52) [4.1 (0.1, 8.1)] | 0.045 | 1.25 (1.07, 1.47) [4.4 (1.2, 7.5)] | 0.006 | 1.06 (0.87, 1.31) [1.3 (-2.9, 5.4)] | 0.549 |
| Oral antidiabetics (if diabetic) | 0.95 (0.57, 1.56) [-1.0 (-11.1, 9.0)] | 0.840 | 1.14 (0.72, 1.77) [2.5 (-6.4, 11.4)] | 0.569 | 1.38 (0.74, 2.64) [6.0 (-5.7, 17.6)] | 0.321 | 0.96 (0.57, 1.61) [-0.9 (-11.3, 9.6)] | 0.868 | 1.11 (0.70, 1.75) [1.9 (-7.1, 11.0)] | 0.669 | 1.31 (0.69, 2.49) [5.0 (-6.8, 16.8)] | 0.407 |
| Insulin (if diabetic) | 1.09 (0.67, 1.78) [1.8 (-8.5, 12.2)] | 0.732 | 1.19 (0.78, 1.86) [3.9 (-5.4, 13.1)] | 0.421 | 0.91 (0.50, 1.65) [-2.0 (-14.4, 10.4)] | 0.753 | 1.08 (0.65, 1.80) [1.7 (-9.1, 12.5)] | 0.762 | 1.22 (0.78, 1.89) [4.3 (-5.2, 13.7)] | 0.389 | 0.96 (0.53, 1.76) [-0.8 (-13.4, 11.8)] | 0.899 |
| **Second follow-up (6.6 years)** |  |  |  |  |  |  |  |  |  |  |  |  |
| Aspirin | 0.98 (0.63, 1.53) [-0.2 (-4.2, 3.8)] | 0.923 | 1.11 (0.76, 1.59) [0.9 (-2.3, 4.1)] | 0.587 | 0.91 (0.58, 1.45) [-0.8 (-5.1, 3.4)] | 0.700 | 1.14 (0.72, 1.80) [1.2 (-2.9, 5.2)] | 0.575 | 1.18 (0.81, 1.71) [1.4 (-1.9, 4.7)] | 0.389 | 0.82 (0.53, 1.30) [-1.9 (-6.5, 2.6)] | 0.403 |
| Anti-P2Y12 | 0.88 (0.57, 1.35) [-1.3 (-5.4, 2.9)] | 0.557 | 1.05 (0.75, 1.48) [0.5 (-3.0, 4.0)] | 0.775 | 1.05 (0.68, 1.61) [0.5 (-3.9, 5.0)] | 0.819 | 0.85 (0.55, 1.32) [-1.6 (-5.8, 2.7)] | 0.471 | 1.03 (0.73, 1.45) [0.3 (-3.2, 3.8)] | 0.871 | 1.10 (0.71, 1.69) [1.0 (-3.6, 5.6)] | 0.672 |
| Statins | 1.01 (0.70, 1.45) [0.1 (-4.9, 5.1)] | 0.970 | 1.56 (1.15, 2.12) [5.4 (1.5, 9.2)] | 0.004 | 1.01 (0.70, 1.47) [0.2 (-5.0, 5.4)] | 0.943 | 1.18 (0.81, 1.71) [2.3 (-2.8, 7.3)] | 0.385 | 1.64 (1.20, 2.23) [6.0 (2.0, 10.1)] | 0.002 | 1.06 (0.73, 1.54) [0.8 (-4.5, 6.1)] | 0.759 |
| ACEi/ARB | 1.32 (0.98, 1.78) [5.8 (-0.3, 11.9)] | 0.064 | 1.36 (1.07, 1.73) [6.4 (1.4, 11.3)] | 0.010 | 0.96 (0.72, 1.30) [-0.8 (-7.3, 5.7)] | 0.812 | 1.21 (0.90, 1.63) [3.9 (-2.0, 9.9)] | 0.198 | 1.17 (0.93, 1.48) [3.2 (-1.6, 8.0)] | 0.190 | 0.94 (0.71, 1.26) [-1.2 (-7.4, 4.9)] | 0.694 |
| Beta-blockers | 1.44 (1.10, 1.90) [8.7 (2.2, 15.3)] | 0.009 | 1.34 (1.07, 1.67) [7.0 (1.6, 12.3)] | 0.010 | 0.86 (0.65, 1.13) [-3.8 (-10.7, 3.1)] | 0.276 | 1.21 (0.92, 1.60) [4.6 (-2.0, 11.3)] | 0.170 | 1.17 (0.94, 1.46) [3.8 (-1.5, 9.1)] | 0.160 | 0.85 (0.64, 1.11) [-4.1 (-10.9, 2.6)] | 0.232 |
| Oral antidiabetics (if diabetic) | 0.90 (0.37, 2.10) [-2.0 (-17.4, 13.4)] | 0.802 | 0.99 (0.45, 2.04) [-0.2 (-13.3, 12.9)] | 0.972 | 0.76 (0.30, 1.92) [-5.3 (-22.8, 12.2)] | 0.554 | 0.78 (0.32, 1.87) [-4.7 (-20.9, 11.6)] | 0.574 | 0.87 (0.41, 1.84) [-2.6 (-15.8, 10.7)] | 0.710 | 0.77 (0.31, 1.94) [-4.8 (-22.0, 12.4)] | 0.581 |
| Insulin (if diabetic) | 0.67 (0.31, 1.43) [-9.2 (-26.7, 8.3)] | 0.303 | 0.84 (0.44, 1.59) [-4.3 (-19.6, 11.1)] | 0.583 | 0.75 (0.32, 1.73) [-6.6 (-26.1, 12.8)] | 0.508 | 0.73 (0.33, 1.61) [-7.2 (-25.5, 11.1)] | 0.440 | 0.84 (0.44, 1.61) [-4.2 (-19.7, 11.3)] | 0.595 | 0.76 (0.33, 1.77) [-6.3 (-25.8, 13.2)] | 0.530 |
| Unadjusted odds ratios, adjusted odds ratios, raw mean difference, and average marginal effect with 95% confidence interval are presented. Model 1 is unadjusted, and model 3 is adjusted for age, sex and traditional cardiovascular risk factors (hypertension, body mass index, smoking status, diabetes, previous myocardial infarction and hypercholesterolemia). Results from model 2 (adjusted for age and sex) are presented in the manuscript. *Abbreviations: OR = odds ratio, RMD = raw mean difference, CI = confidence interval* | | | | | | | | | | | | |

**Supplementary Table 5.** Unadjusted (model 1) and adjusted (model 3) associations of achievement of secondary prevention with education levels

|  | **Model 1**  **(unadjusted)** | | | | | | **Model 3**  **(adjusted for age, sex and traditional risk factors)** | | | | | |
| --- | --- | --- | --- | --- | --- | --- | --- | --- | --- | --- | --- | --- |
|  | EL 1 vs. EL4 | | EL2 vs. EL4 | | EL3 vs. EL4 | | EL 1 vs. EL4 | | EL2 vs. EL4 | | EL3 vs. EL4 | |
|  | Unadj OR (95%CI)  [RMD (95% CI)] | p-value | Unadj. OR (95%CI)  [RMD (95% CI)] | p-value | Unadj. OR (95%CI)  [RMD (95% CI)] | p-value | Adj. OR (95%CI)  [AME (95% CI)] | p-value | Adj. OR (95%CI)  [AME (95% CI)] | p-value | Adj. OR (95%CI)  [AME (95% CI)] | p-value |
| **First follow-up (1 year)** |  |  |  |  |  |  |  |  |  |  |  |  |
| LDL-C <1.8mmol/l | 0.85 (0.64, 1.12) [-2.8 (-7.6, 2.0)] | 0.254 | 0.84 (0.67, 1.05) [-3.1 (-7.1, 0.9)] | 0.122 | 0.88 (0.66, 1.18) [-2.1 (-7.1, 2.8)] | 0.402 | 0.90 (0.68, 1.20) [-1.7 (-6.6, 3.1)] | 0.483 | 0.87 (0.69, 1.09) [-2.4 (-6.4, 1.5)] | 0.222 | 0.97 (0.73, 1.29) [-0.5 (-5.5, 4.4)] | 0.831 |
| HbA1c <7% (if diabetic) | 0.74 (0.36, 1.48) [-7.3 (-24.1, 9.4)] | 0.396 | 0.64 (0.34, 1.18) [-10.9 (-25.8, 3.9)] | 0.158 | 1.66 (0.69, 4.20) [11.0 (-7.7, 29.6)] | 0.264 | 0.67 (0.32, 1.39) [-9.7 (-27.2, 7.8)] | 0.281 | 0.66 (0.35, 1.23) [-10.3 (-25.3, 4.7)] | 0.187 | 1.76 (0.72, 4.28) [12.0 (-6.3, 30.3)] | 0.214 |
| Systolic blood pressure <140mmHg | 0.80 (0.64, 1.01) [-4.6 (-9.5, 0.2)] | 0.059 | 1.12 (0.92, 1.34) [2.1 (-1.5, 5.8)] | 0.251 | 0.94 (0.74, 1.20) [-1.3 (-6.1, 3.6)] | 0.609 | 0.94 (0.75, 1.18) [-1.3 (-6.0, 3.4)] | 0.598 | 1.21 (1.01, 1.45) [3.7 (0.1, 7.3)] | 0.040 | 0.88 (0.70, 1.10) [-2.8 (-7.6, 2.0)] | 0.254 |
| Weight reduction ≥5% (if overweight or obese) | 0.75 (0.55, 1.00) [-4.7 (-9.3, 0.0)] | 0.052 | 0.87 (0.69, 1.11) [-2.2 (-6.2, 1.7)] | 0.260 | 1.16 (0.86, 1.56) [2.7 (-2.7, 8.0)] | 0.329 | 0.68 (0.51, 0.92) [-6.0 (-10.7, -1.3)] | 0.013 | 0.86 (0.68, 1.09) [-2.5 (-6.5, 1.5)] | 0.213 | 1.14 (0.85, 1.53) [2.5 (-2.9, 7.8)] | 0.366 |
| Smoking cessation (if smoker) | 0.65 (0.49, 0.87) [-10.7 (-17.9, -3.4)] | 0.004 | 0.80 (0.63, 1.03) [-5.5 (-11.6, 0.6)] | 0.079 | 0.83 (0.61, 1.13) [-4.7 (-12.4, 2.9)] | 0.227 | 0.71 (0.53, 0.95) [-8.5 (-15.8, -1.2)] | 0.023 | 0.81 (0.64, 1.04) [-5.1 (-11.2, 0.9)] | 0.098 | 0.84 (0.62, 1.15) [-4.2 (-11.8, 3.4)] | 0.278 |
| Alcohol consumption reduction (if daily alcohol consumer) | 1.35 (0.92, 1.97) [6.6 (-1.7, 15.0)] | 0.120 | 1.36 (1.01, 1.85) [6.9 (0.3, 13.4)] | 0.046 | 1.29 (0.87, 1.91) [5.6 (-3.0, 14.3)] | 0.198 | 1.15 (0.78, 1.69) [3.1 (-5.4, 11.6)] | 0.472 | 1.26 (0.93, 1.71) [5.2 (-1.5, 12.0)] | 0.134 | 1.17 (0.79, 1.72) [3.5 (-5.1, 12.1)] | 0.426 |
| **Second follow-up (6.6 years)** |  |  |  |  |  |  |  |  |  |  |  |  |
| LDL-C <1.8mmol/l | 0.57 (0.39, 0.83) [-9.6 (-15.9, -3.3)] | 0.004 | 0.75 (0.56, 1.00) [-5.3 (-10.7, 0.0)] | 0.047 | 0.64 (0.44, 0.93) [-7.8 (-14.3, -1.3)] | 0.022 | 0.63 (0.43, 0.92) [-7.9 (-14.3, -1.6)] | 0.017 | 0.83 (0.62, 1.11) [-3.4 (-8.8, 1.9)] | 0.203 | 0.72 (0.49, 1.05) [-5.8 (-12.3, 0.7)] | 0.085 |
| HbA1c <7% (if diabetic) | 1.15 (0.49, 2.72) [3.4 (-17.7, 24.4)] | 0.754 | 0.76 (0.37, 1.60) [-6.4 (-24.0, 11.1)] | 0.469 | 1.23 (0.49, 3.10) [5.0 (-17.7, 27.7)] | 0.665 | 0.88 (0.36, 2.13) [-3.2 (-24.8, 18.4)] | 0.770 | 0.73 (0.35, 1.54) [-7.5 (-25.5, 10.5)] | 0.408 | 1.40 (0.56, 3.47) [8.3 (-14.3, 30.9)] | 0.472 |
| Systolic blood pressure <140mmHg | 0.80 (0.52, 1.24) [-4.9 (-14.7, 4.9)] | 0.324 | 1.07 (0.75, 1.53) [1.5 (-6.2, 9.2)] | 0.704 | 0.77 (0.49, 1.19) [-5.9 (-15.9, 4.0)] | 0.241 | 0.89 (0.57, 1.38) [-2.7 (-12.5, 7.2)] | 0.594 | 1.19 (0.83, 1.69) [3.7 (-3.9, 11.2)] | 0.340 | 0.73 (0.47, 1.12) [-7.4 (-17.3, 2.6)] | 0.147 |
| Weight reduction ≥5% (if overweight or obese) | 1.39 (0.84, 2.32) [5.8 (-3.1, 14.7)] | 0.205 | 1.40 (0.92, 2.16) [5.9 (-1.2, 13.0)] | 0.120 | 1.09 (0.62, 1.90) [1.4 (-7.8, 10.7)] | 0.762 | 0.94 (0.57, 1.56) [-1.1 (-10.2, 8.0)] | 0.816 | 1.05 (0.70, 1.59) [0.9 (-6.7, 8.5)] | 0.813 | 0.96 (0.56, 1.65) [-0.7 (-10.4, 9.1)] | 0.893 |
| Smoking cessation (if smoker) | 0.81 (0.52, 1.25) [-5.2 (-16.1, 5.6)] | 0.343 | 0.82 (0.57, 1.18) [-5.0 (-14.0, 4.1)] | 0.283 | 0.59 (0.37, 0.92) [-13.2 (-24.4, -2.0)] | 0.022 | 0.84 (0.54, 1.30) [-4.4 (-15.3, 6.5)] | 0.431 | 0.82 (0.57, 1.18) [-5.0 (-14.1, 4.0)] | 0.279 | 0.58 (0.37, 0.91) [-13.6 (-24.7, -2.4)] | 0.018 |
| Alcohol consumption reduction (if daily alcohol consumer) | 1.81 (1.11, 2.98) [14.3 (2.6, 26.0)] | 0.018 | 1.42 (0.94, 2.14) [8.2 (-1.3, 17.6)] | 0.095 | 1.08 (0.61, 1.88) [1.7 (-11.0, 14.4)] | 0.789 | 1.33 (0.81, 2.20) [6.9 (-5.2, 18.9)] | 0.265 | 1.21 (0.80, 1.82) [4.5 (-5.1, 14.2)] | 0.361 | 0.92 (0.53, 1.59) [-2.0 (-14.7, 10.7)] | 0.754 |
| Unadjusted odds ratios, adjusted odds ratios, raw mean difference, and average marginal effect with 95% confidence interval are presented. Model 1 is unadjusted, and model 3 is adjusted for age, sex and traditional cardiovascular risk factors (hypertension, body mass index, smoking status, diabetes, previous myocardial infarction and hypercholesterolemia). Results from model 2 (adjusted for age and sex) are presented in the manuscript. *Abbreviations: OR = odds ratio, RMD = raw mean difference, CI = confidence interval* | | | | | | | | | | | | |
